# Whole-Brain Functional Network Connectivity Abnormalities in Affective and Non-Affective Early Phase Psychosis

**DOI:** 10.3389/fnins.2021.682110

**Published:** 2021-06-18

**Authors:** Zening Fu, Armin Iraji, Jing Sui, Vince D. Calhoun

**Affiliations:** ^1^Tri-Institutional Center for Translational Research in Neuroimaging and Data Science (TReNDS), Georgia State University, Georgia Institute of Technology, Emory University, Atlanta, GA, United States; ^2^Chinese Academy of Sciences (CAS) Centre for Excellence in Brain Science and Intelligence Technology, University of Chinese Academy of Sciences, Beijing, China; ^3^Department of Psychiatry, School of Medicine, Yale University, New Haven, CT, United States; ^4^Department of Psychology and Computer Science, Neuroscience Institute and Physics, Georgia State University, Atlanta, GA, United States; ^5^Department of Electrical and Computer Engineering, Georgia Institute of Technology, Atlanta, GA, United States

**Keywords:** early phase psychosis, non-affective psychosis, affective psychosis, functional network connectivity (FNC), dynamic functional network connectivity (dFNC), graphic measure

## Abstract

Psychosis disorders share overlapping symptoms and are characterized by a wide-spread breakdown in functional brain integration. Although neuroimaging studies have identified numerous connectivity abnormalities in affective and non-affective psychoses, whether they have specific or unique connectivity abnormalities, especially within the early stage is still poorly understood. The early phase of psychosis is a critical period with fewer chronic confounds and when treatment intervention may be most effective. In this work, we examined whole-brain functional network connectivity (FNC) from both static and dynamic perspectives in patients with affective psychosis (PAP) or with non-affective psychosis (PnAP) and healthy controls (HCs). A fully automated independent component analysis (ICA) pipeline called “Neuromark” was applied to high-quality functional magnetic resonance imaging (fMRI) data with 113 early-phase psychosis patients (32 PAP and 81 PnAP) and 52 HCs. Relative to the HCs, both psychosis groups showed common abnormalities in static FNC (sFNC) between the thalamus and sensorimotor domain, and between subcortical regions and the cerebellum. PAP had specifically decreased sFNC between the superior temporal gyrus and the paracentral lobule, and between the cerebellum and the middle temporal gyrus/inferior parietal lobule. On the other hand, PnAP showed increased sFNC between the fusiform gyrus and the superior medial frontal gyrus. Dynamic FNC (dFNC) was investigated using a combination of a sliding window approach, clustering analysis, and graph analysis. Three reoccurring brain states were identified, among which both psychosis groups had fewer occurrences in one antagonism state (state 2) and showed decreased network efficiency within an intermediate state (state 1). Compared with HCs and PnAP, PAP also showed a significantly increased number of state transitions, indicating more unstable brain connections in affective psychosis. We further found that the identified connectivity features were associated with the overall positive and negative syndrome scale, an assessment instrument for general psychopathology and positive symptoms. Our findings support the view that subcortical-cortical information processing is disrupted within five years of the initial onset of psychosis and provide new evidence that abnormalities in both static and dynamic connectivity consist of shared and unique features for the early affective and non-affective psychoses.

## Introduction

Ranked as the third most disabling condition after quadriplegia and dementia, psychosis is characterized as severer disruptions to the information processing in the brain that affect people’s thoughts and perceptions (Amador and David, 1999; Arciniegas, 2015). Psychotic disorders, such as schizophrenia, bipolar disorder, major depression disorder, and some other forms of psychoses with the presence of hallucinations and delusions, have become a major public health problem that incurs an enormous economic burden worldwide (World Health Organization, 2001). In the United States, around 3 in 100 people have a psychosis in their lives, and approximately 100,000 young people experience this condition every year. Over the past decade, growing evidence has shown that psychosis is associated with disruptions of the coordinated functioning of distributed brain regions (Friston, 1998, 1999; Canuet et al., 2011; Khadka et al., 2013). Such functional disconnections are typically investigated by measuring functional connectivity (i.e., temporal coherence) between fluctuations in low-frequency blood oxygen level-dependent (BOLD) signal in resting-state functional magnetic imaging (rs-fMRI) data (Dandash et al., 2014; Palaniyappan and Liddle, 2014; Satterthwaite and Baker, 2015). Functional abnormalities have been identified in multiple brain systems involving default mode, subcortical, cerebellar, and sensorimotor networks (Mamah et al., 2013; Baker et al., 2014; Palaniyappan and Liddle, 2014; Wang et al., 2020).

Despite the consistent connectivity abnormalities across studies, it is still unclear if the functional brain manifestations are present in early psychosis. Early psychosis (EP), also known as first-episode psychosis, is within five years of the initial onset of psychotic symptoms (American Psychological Association [APA], 2013). EP is a critical period of illness when there are fewer confounds, such as prolonged medication exposure and chronicity. Early intervention is also important for people with psychosis that would lead to more effective treatment (Harrigan et al., 2003; Bird et al., 2010). To advance the new understanding of functional connectivity in psychosis, several investigations of functional connectivity have been conducted in EP data. These studies have revealed increased default mode network-salience network/cerebellum connectivity, increased somatomotor-thalamic connectivity, and decreased pre-frontal-cortex-thalamic connectivity in early-stage psychosis patients, some of which shared overlapping patterns with chronic psychosis (Carhart-Harris et al., 2013; Woodward and Heckers, 2016; Mallikarjun et al., 2018). However, recent work has challenged the “static” assumption of connectivity, which cannot capture the temporal changes in functional connectivity that may be affected in psychosis (Keshavan et al., 2014; Cannon, 2015). Increasing evidence has shown that the functional connectivity is highly dynamic rather than static, especially within the resting-state when mental activity is unconstrained, and such dynamic characteristics are not spurious (Hutchison et al., 2013; Allen et al., 2014, 2018; Lurie et al., 2020). Leveraging this new understanding of functional connectivity, recent studies have investigated the rapid changes in functional connectivity in psychosis data, showing both common and distinct dynamic brain abnormalities between affective and non-affective psychoses (Damaraju et al., 2014; Rashid et al., 2014; Fu et al., 2018; Zhi et al., 2018; Iraji et al., 2019). Yet, there is still little research on the dynamic functional connectivity in EP (Mennigen et al., 2018, 2019), especially between early phase affective and non-affective psychoses. The comprehensive investigation of functional connectivity in EP may improve our understanding of shared and unique brain abnormalities in early phase affective and non-affective psychoses and the brain manifestations across the psychosis continuum.

In this work, we applied a fully automated pipeline called Neuromark to fMRI data of patients with early-phase affective and non-affective psychoses collected by the Human Connectome Project. This pipeline combines Neuromark templates, independent component analysis (ICA), a sliding window approach, k-means clustering, and graph analysis to capture comprehensive functional connectivity characteristics from both static and dynamic perspectives (Du et al., 2020). We hypothesize that the EP patients will show abnormalities in both static and dynamic functional connectivity that are similar to those observed in chronic psychosis. Such similarities will reflect manifestations of psychosis-related pathophysiology. We also aim to explore the shared and distinct brain changes between affective and non-affective psychoses in the early stage and their potential associations with the severity of symptoms.

## Materials and Methods

### Participants

The dataset analyzed in this study is from the Human Connectome Project for Early Psychosis (HCP-EP), which acquires high-quality data as part of the original HCP data^1^. This project focused on early-phase psychosis, within the first five years of the onset of psychotic symptoms. The release 1.0 of the HCP-EP data is provided on NDA^2^ which includes 183 subjects collected from 4 clinical recruitment sites (Indiana University [IU], Beth Israel Deaconess Medical Center – Massachusetts Mental Health Center [BIDMC], McLean Hospital [MH] and Massachusetts General Hospital [MGH]). Medically stable male and female subjects with a confirmed psychiatric diagnosis and HC subjects were enrolled in the HCP-EP study. The diagnosis of affective and non-affective psychosis is based on DSM-V (American Psychological Association [APA], 2013). The PAP group included subjects with the diagnosis of major depression with psychosis (single and recurrent episodes) or bipolar disorder with psychosis (including most recent episode depressed and manic types) with onset within five years prior to study entry. The PnAP group included subjects with the diagnosis of schizophrenia, schizophreniform, schizoaffective, psychosis NOS, delusional disorder, or brief psychotic disorder with onset within the past five years prior to study entry. The HC group required subjects do not meet the criteria for bipolar and related disorders, major depressive disorder, schizophrenia, anxiety disorder, and other psychotic disorders. More details of the inclusion criteria can be found in (See text footnote 2). The image data were scanned using Siemens MAGNETOM Prisma 3T scanners with a multiband sequence and a 32/64-channel head coil. The rs-fMRI data is with 2 mm isotropic resolution, multi-band acceleration factor of 8, repetition time (TR) = 720 ms and was acquired twice with posterior-anterior (PA) and anterior-posterior (AP) phase encoding. More details of the imaging protocols can be found on the NDA website (See text footnote).

### Preprocessing

The fMRI data were preprocessed using a combination of FSL and statistical parametric mapping (SPM12) under the MATLAB 2019 environment. Before motion correction, a distortion field was calculated from the PA and AP phase-encoded field maps by the topup/FSL algorithm to correct for intensity and geometric distortions. Then a rigid body motion correction was performed using SPM to correct the head motions in fMRI scans. After that, the fMRI data were subsequently normalized to the standard Montreal Neurological Institute (MNI) space using an echo-planar imaging (EPI) template and were slightly resampled to 3 × 3 × 3 mm isotropic voxels. The resampled fMRI images were finally smoothed using a Gaussian kernel with a full width at half maximum (FWHM) = 6 mm. Since the dynamic functional connectivity analysis is sensitive to the data quality, we did the quality control (QC) by selecting subjects with functional data providing near full brain successful normalization for further analysis (see Supplementary Materials, section Subject Selection by Comparing Group Mask with Individual Mask Fu et al., 2019b). This yielded in total 170 subjects, consisting of 57 HCs, 32 PAPs, and 81 PnAPs. Most of the patients have been collected the positive and negative syndrome scale (PANSS) (Kay et al., 1987), which includes 30 items that assess psychopathology and positive symptoms in patients with psychosis. The HCP-EP dataset also recorded lifetime antipsychotic medication dosage as Chlorpromazine (CPZ) equivalents using the Gardner approach (Gardner et al., 2010), full intelligence quotient (IQ) using the Wechsler Abbreviated Scale of Intelligence, Second Edition (WASI-II), Young Mania Rating Scale (YMRS) (Young et al., 1978) and Montgomery-Asberg Depression Rating Scale (MADRS) (Montgomery and Asberg, 1979). Details of these demographics for the included participants can be found in Table 1.

**TABLE 1:** Demographics and PANSS of HCP-EP Dataset.

Characteristics	HCP-EP		
	
	HCs (n = 57)	PAPs (n = 32)	PnAPs (n = 81)
Age (years)	24.794.12	23.964.41	22.343.54
Gender (female/male)	20/37	19/13	26/55
Site (IU/BIDMC/MGH/MH)	25/8/11/13	6/7/2/17	50/14/5/12
CPZ (29 subjects recorded)	NA	366.67208.17	423.08229.88
IQ (WASI-II) (157 subjects recorded)	115.9610.78	106.6114.80	98.7018.01
YMRS (72 subjects recorded)	NA	5.975.76	5.795.06
MADRS (69 subjects recorded)	NA	9.588.12	9.707.11
PANSS Total	NA	41.629.68	53.339.81

*HCP-EP = Human Connectome Project for Early Psychosis; HC = Healthy controls; PAPs = patients with affective psychosis; PnAPs = patients with non-affective psychosis; CPZ = Chlorpromazine; IQ = intelligence quotient; YMRS = Young Mania Rating Scale; MADRS = Montgomery-Asberg Depression Rating Scale; PANSS = Positive and Negative Syndrome Scale.*

### Schematic Diagram of Neuromark Pipeline

**FIGURE 1:**
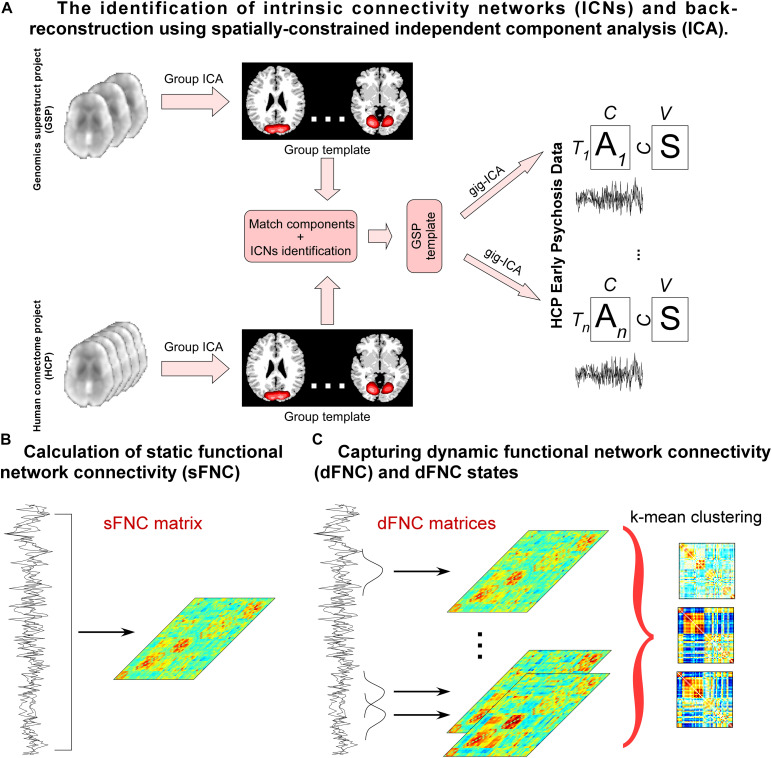
Flowchart of capturing whole-brain connectivity features. **(A)** group ICA is performed on two independent healthy controls datasets and the estimated independent components (ICs) are matched by the spatial correlation. Matched ICs are identified as intrinsic connectivity networks (ICNs) according to their spatial maps and the ICNs are used as spatial templates to calculate components for the HCPEP data. **(B)** Pearson correlation coefficients are calculated using the TCs across the whole scans. **(C)** A sliding window approach is used to estimate dFNC. K means clustering is performed on the dFNC estimates. State occurrences and transitions are calculated, and graphic measure is calculated for each state.

A flowchart of the Neuromark pipeline is provided in Figure 1. Neuromark is a robust ICA-based analysis pipeline that can capture corresponding functional network features while retaining more single-subject variability (Du et al., 2020). This pipeline has been successfully applied to multiple studies and identified a wide range of connectivity abnormalities in numerous brain diseases (Fu et al., 2019a, 2020; Li et al., 2020; Tu et al., 2020). In this pipeline, group ICA was first performed on two large healthy control datasets to construct spatial network (component) priors. Then based on the network priors, a spatially-constrained ICA algorithm was applied to estimate spatial maps and time-courses (TCs) for each subject from the HCP-EP dataset. Static functional network connectivity (sFNC) is calculated by the Pearson correlation coefficient and dynamic functional network connectivity (dFNC) is estimated via a sliding window approach. After that, k-means clustering is performed on the dFNC features. dFNC state characteristics are calculated, and graph analysis is performed in a state-based manner.

### Static FNC and Dynamic FNC

Before measuring the sFNC and dFNC, four post-processing procedures were performed on the extracted TCs of each subject to denoise the data (Allen et al., 2014): (1) detrending linear, quadratic, and cubic trends; (2) despiking temporal outlier; (3) low-pass filtering with a cutoff frequency of 0.15 Hz and (4) regressing out six head motion parameters and their temporal derivatives.

sFNC was calculated using the Pearson correlation coefficients between TCs, resulting in an sFNC matrix (C × C, C is the number of ICNs selected from the templates) for each subject. Diagnosis effects (1 = HC, 2 = PnAP, 3 = PAP) were examined in each sFNC by N-way analysis of variance (N-ANOVA), using age, gender, and site as the confounding effects. If the diagnosis effect is significant, a *post hoc* general linear model (GLM) was conducted to examine the group difference between pairs of groups, controlling for age, gender, and site.

dFNC was estimated via a sliding window approach. We used a tapered window to localize the data, which is created by convolving a rectangle (width = 40 TRs = 28.8s) with a Gaussian (σ = 3 TRs). The window was slid by 1 TR, resulting in 361 time-windows. Therefore, for each subject, the dFNC estimate is a C × C × T (T = 361) array representing the dynamic changes in brain connectivity as functions of time (Allen et al., 2014). A k means clustering method was applied to the dFNC estimates to identify reoccurring connectivity patterns across time and subjects, referred as dynamic brain states. The optimal number of clusters was *k* = 3, which is determined by the elbow criterion, defined as the ratio of within-cluster distance to between-cluster distance. We calculated the fractional rate by dividing the number of the total windows by the number of windows assigned to each state and the number of total transitions between states as the measures of dynamic characteristics. N-ANOVA was performed to examine the diagnosis effect. If the effect is significant, a *post hoc* GLM was conducted to probe the difference between pairs of groups. The statistical results were corrected using false discovery rate (FDR) correction (Benjamini and Hochberg, 1995).

### Dynamic Graphic Measure

To further explore if EP patients show disruptions of information transmission in specific brain networks, we investigated the local efficiency, a graphic measure within each dynamic brain state. The local efficiency is the average inverse shortest path length in the network computed on the neighborhood of the node, reflecting whether a node is well connected to the global network. The local efficiency of each ICN was estimated using the dFNC estimates via the brain connectivity toolbox^3^. We averaged the local efficiency across windows assigned to each state and performed N-ANOVA and further GLM to examine the diagnosis effect, controlling for age, gender, and site. We also replicated the results by adding full IQ as one covariate and the results are provided in the Supplementary Materials, section Replication of Findings by Adding Full IQ as one Covariate.

### Associations Between FNC Features and PANSS

The potential associations between FNC features (including pair-wise sFNC, fractional rate of state, number of state transitions and local efficiency of state) and PANSS were investigated by the GLM, controlling for age, gender, and site. Note that, PANSS was only available for patients with early psychosis. To prevent the potential confounding effect induced by the difference between affective/non-affective psychosis, we further repeated this analysis by adding group label as one of the covariates in the GLM. We also performed the correlation analysis between FNC features and YMRS/MADRS to investigate more potential associations with the clinical profiles. The results are provided in the Supplementary Materials, section Associations with Young Mania Rating Scale (YMRS) and Montgomery-Asberg Depression Rating Scale (MADRS).

## Results

### Brain Parcellation and Static FNC

**FIGURE 2:**
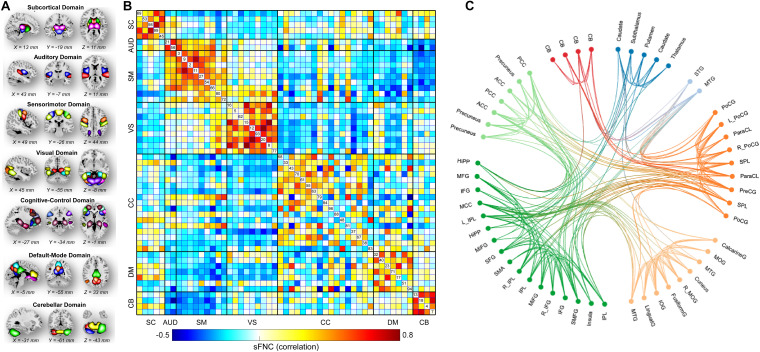
Spatial maps of the identified ICNs and sFNC matrix. **(A)** 53 ICNs were identified and sorted into seven resting-state functional domains. Each color represents a single ICN. **(B)** Averaged sFNC matrix across participants. **(C)** The functional connectivity profile of the averaged sFNC matrix.

The brain was parcellated into 53 ICNs using Neuromark (Du et al., 2020). More details on the Neuromark pipeline such as the cohorts used to construct spatial priors can be found in Supplementary Materials, section Neuromark Framework and (Du et al., 2020). The 53 ICNs were arranged into seven functional domains according to their anatomic and functional prior knowledge. The seven functional domains were displayed in Figure 2A, including subcortical (SC, 5 ICNs), auditory (AUD, 2 ICNs), visual (VS, 9 ICNs), sensorimotor (SM, 9 ICNs), cognitive-control (CC, 17 ICNs), default-mode (DM, 7 ICNs), and cerebellar domains (CB, 4 ICNs). After the additional post-processing procedures on the TCs, we calculated a 53 × 53 correlation matrix for each subject as the measure of sFNC. As seen from Figure 2B, there is strong within domain connectivity across subjects, especially for SC, sensory domains, DM, and CB.

**FIGURE 3:**
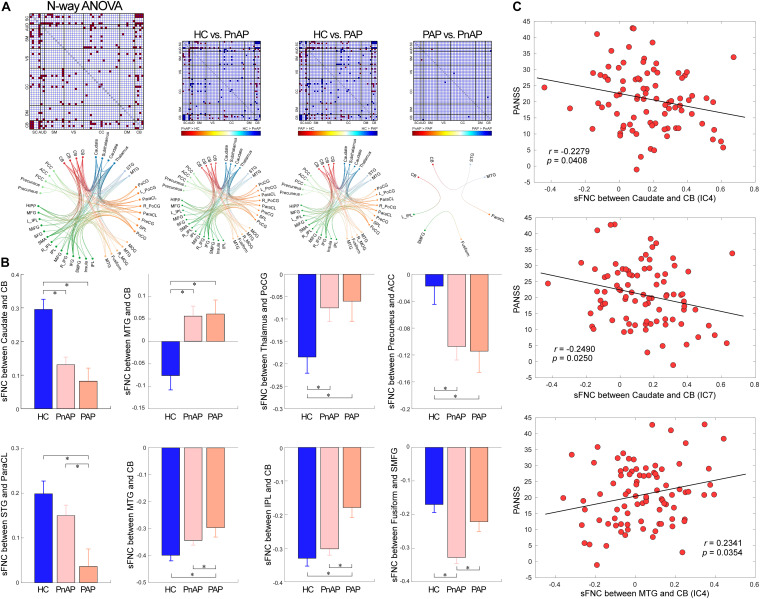
Findings of sFNC analysis. **(A)** N-way ANOVA results and pair-wise group comparisons (HC vs. PnAP, HC vs. PAP, PnAP vs. PAP). The FNC showed significant difference in the ANOVA test are marked in red. For pair-wise comparison results, significantly different FNC are marked in red or blue to indicate the direction of difference (e.g., HC > PnAP or PnAP > HC). The Functional connectivity profile of the significant FNC is displayed by circular graph. **(B)** Exemplars of abnormal sFNC between groups. Data are presented as mean values ± standard error of mean (SEM). PnAP and PAP showed shared and unique abnormalities. **(C)** Correlations between abnormal sFNC and PANSS total score. ^∗^Significance *p* < 0.05, FDR corrected.

According to the results displayed in Figures 3A,B, compared with HCs, PnAP and PAP showed similarly altered sFNC across the whole brain (*p* < 0.05, FDR corrected). The major abnormalities concentrate on SC, SM, and CB. Specifically, patient groups showed reduced connectivity between the caudate/subthalamus/thalamus and the cerebellum, reduced connectivity between the thalamus and the caudate/subthalamus, and increased connectivity between the caudate/subthalamus/thalamus and ICNs within SM (e.g., postcentral gyrus [PoCG] and Paracentral lobule [ParaCL]). PnAP and PAP also showed unique sFNC abnormalities (*p* < 0.05, FDR corrected). For example, PAP showed decreased connectivity between the supper temporal gyrus (STG) and the ParaCL, and decreased connectivity between the cerebellum and the middle temporal gyrus (MTG)/inferior parietal lobule (IPL), while these abnormalities cannot be observed in PnAP. On the other hand, PnAP showed specifically increased negative connectivity (more negative) between the fusiform and the superior medial frontal gyrus (SMFG). Moreover, we found that within the patient cohorts (*n* = 113), the shared abnormal sFNC showed correlations with the spectrum of severity, as indexed by the PANSS total score. The results in Figure 3C showed that the sFNC between caudate and cerebellum was negatively correlated with PANSS (*r* = −0.2279, *p* = 0.0408, uncorrected), the sFNC between caudate and cerebellum was negatively correlated with PANSS (*r* = −0.2490, *p* = 0.0250, uncorrected), and the sFNC between MTG and cerebellum was positively correlated with PANSS (*r* = 0.2341, *p* = 0.0354, uncorrected).

### Dynamic FNC Characteristics

**FIGURE 4:**
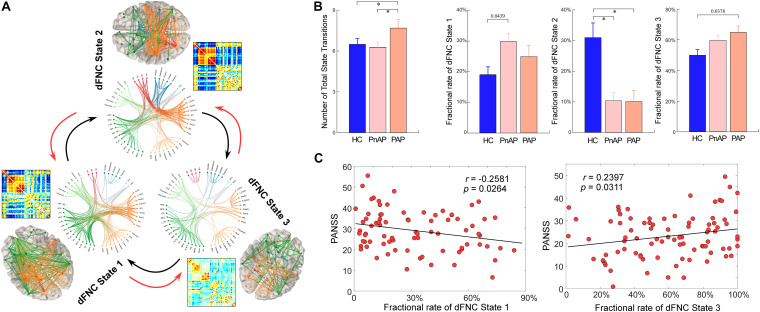
Group differences in dynamic characteristics of dFNC states. **(A)** Functional connectivity profile of each brain state: top 200 FNC with connectivity strength >0.2 in each state, representing the strongest functional-relationships between brain regions. **(B)** Group differences in the fractional rate and state transitions. Bars represent the mean of occurrences and error bars represent the SEM. **(C)** Scatter plots of correlations between dynamic characteristics and PANSS total score. ^∗^Significance *p* < 0.05, FDR corrected.

Figure 4A displays the clustering results with the number of states as 3. The circles display the functional profile of brain states which retains the strong connectivity (absolute value >0.2) and the brains display the corresponding connectivity patterns mapping to the ICBM152 brain template. One sparsely connected state (state 3) has more frequent occurrence while two strong connectivity states have less frequent occurrence (state 1 and state 2). States 1 and 2 have strongly positive connectivity within the sensory domains (AUD, SM, and VS) and negative connectivity between the sensory domains and DM/CB domains. These states also show different connectivity patterns. For example, state 1 shows negative connectivity between SM and CC domains and weak connectivity between SC and sensory domains. In contrast, state 2 has strongly negative connectivity between SC and sensory domains, and strongly positive connectivity between SM and CC domains. There are fewer negative and positive connectivity patterns in state 3, which only shows some homogeneity within functional domains. This state accounts for the most windows and resembles the sFNC patterns (compare to Figure 2B).

We calculated two dynamic characteristics of dFNC, fractional rate of the state, which is the proportion of time spent in each state, and the number of transitions, which is the measure of the total number of switches between dFNC states. In total 152 (89.41%, 152/170) subjects have entered dFNC state 1, among which 46 (80.70%, 46/57) are HCs, 31 (96.88%, 31/32) are PAPs and 75 (92.59%, 75/81) are PnAPs. 71 (41.76%, 71/170) subjects have dFNC state 2, among which 34 (59.65%, 34/57) are HCs, 12 (37.50%, 12/32) are PAPs and 25 (30.86%, 25/81) are PnAPs. 169 (99.41%) subjects have entered dFNC state 3, among which 56 (98.25%, 56/57) are HCs, 32 (100%, 32/32) are PAPs and 81 (100%, 81/81) are PnAPs. The group comparison results are displayed in Figure 4B. Compared with HCs, both patient groups had a significantly lower fractional rate in state 2 (*p* < 0.05, FDR corrected). State 1 and state 3 did not show different fractional rate between groups, but their combined fractional rate was significantly higher in the patient groups (results are provided in the Supplementary Materials). Moreover, unlike PnAP, PAP showed a significantly increased number of state transitions during the resting-state (*p* < 0.05, FDR corrected). Further correlation analysis results in Figure 4C showed that the fractional rates of State 1 and 3 were significantly correlated with PANSS total score (*r* = −0.2581, *p* = 0.0264, uncorrected; *r* = 0.2397, *p* = 0.0311, uncorrected), indicating that more severe symptoms may result in a lower occurrence rate of state 1but a higher occurrence rate of state 3.

### Reduced Efficiency of Dynamic States

**FIGURE 5:**
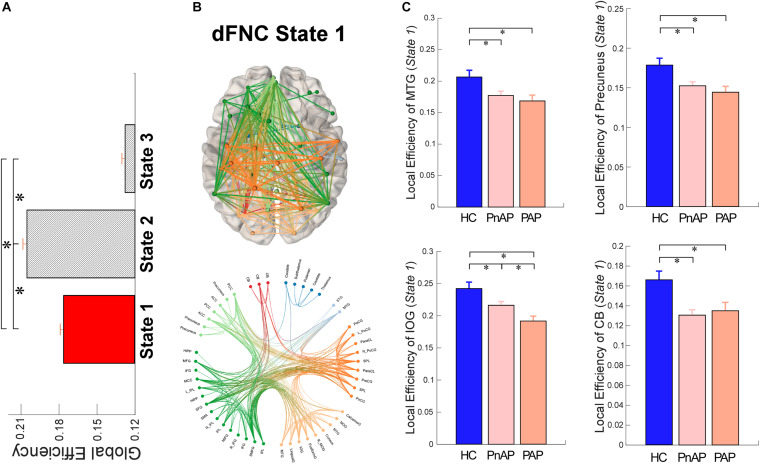
Local Efficiency of dFNC states. **(A)** Comparisons of averaged local efficiency among brain states. **(B)** Functional connectivity profile of state 1 and its connectivity mapping to the ICBM152 brain template. **(C)** Group differences in local efficiency of ICNs within state 1. Bars represent the mean of occurrences and error bars represent the SEM. ^∗^Significance *p* < 0.05, FDR corrected.

To investigate the topological organizations of the brain states and compare them between groups, we applied the graph-theory analysis and calculated a well-established and widely validated measure, local efficiency. The results of graph analysis were displayed in Figure 5. We observed that brain states showed significantly different averaged efficiency, suggesting that the average parallel information transfer in the brain networks is different among brain states. More interestingly, we found that within state 1, both patient groups showed significantly lower local efficiency in the MTG, precuneus, inferior occipital gyrus (IOG), and cerebellum (*p* < 0.05, FDR corrected). In addition, PAP had even lower local efficiency in the IOG within state 1 (*p* < 0.05, FDR corrected), indicating a substantial diminished of information transmission in affective psychosis.

## Discussion

### Abnormalities of Static FNC in Early Psychosis

Our results showed that both PnAP and PAP in the early phase are associated with alterations of static functional connectivity mainly involving the subcortical, sensory, cognitive-control, and cerebellar domains. These findings are in line with prior neuroimaging results in chronic and early psychosis. Thalamus is a well-known region with abnormalities in psychotic disorders (Andreasen, 1997; Hazlett et al., 1999; Byne et al., 2009). Thalamocortical connectivity abnormalities have been widely reported in both chronic and early stages of psychosis and are associated with cognitive impairments caused by the psychosis (Carhart-Harris et al., 2013; Rashid et al., 2014; Woodward and Heckers, 2016; Bergé et al., 2020). Similarly, impairments in sensorimotor, visual, and auditory processes are key symptoms of psychosis (Javitt, 2009; Javitt and Freedman, 2015; Kaufmann et al., 2015; Mallikarjun et al., 2018) and their connectivity abnormalities have been well-documented in previous literature (Damaraju et al., 2014; Fu et al., 2018; Krukow et al., 2018; Zhang et al., 2020; Iraji et al., 2021). Cerebellum and cognitive-control networks that receive information from multiple brain systems are also important for information processing (Leiner et al., 1993; Schutter and van Honk, 2006; Baker et al., 2014) and the disruptions of their interactions are supposed to be shared features in affective and non-affective psychosis, associating with the diseases-related cognitive impairments (Bernard et al., 2014; Woodward and Heckers, 2016; Shinn et al., 2017). The results in our study are highly consistent and also extend findings of pre-mentioned studies by comprehensively investigating whole-brain functional connectivity of affective and non-affective early phase psychosis, respectively. Both affective and non-affective psychosis patients in their early-stage share homogeneous connectivity abnormalities within those common functional domains, suggesting that the connectivity among subcortical, sensory, and cerebellar domains might be associated with the same pathophysiological mechanism of both psychosis groups that underly their overlapping clinical features and symptoms. This hypothesis is further supported by our additional correlation analysis which showed that the shared FNC abnormalities are correlated with the psychotic symptoms, indexed by the subjects’ PANSS total score.

Despite the similarity, PnAP and PAP also showed specific abnormalities in sFNC. PAP group had significantly reduced functional connectivity (less positive) between the STG and the ParaCL and reduced functional connectivity (less negative) between the MTG and the cerebellum. In contrast, PnAP showed weaker changes in these sFNC with less significance. The temporal network-related connections have been previous identified as uniquely affected in affective psychosis, not in non-affective psychosis (Meda et al., 2012). This network and its connectivity with other mood-regulatory regions are likely linked to emotional regulation and memory, which are significantly affected in chronic affective psychosis (Pavuluri et al., 2007; Pavuluri and Passarotti, 2008). Our results provide further evidence that affective psychosis affects these network connections within the early onset of clinical psychosis when there are less prolonged medication exposure and fewer chronicity effects. PnAP group also had unique changes in resting-state connectivity, reflecting by significantly increased sFNC (more negative) between the fusiform and the SMFG. Previous studies have suggested increased local activations (measured by the amplitude of low-frequency fluctuations [ALFF]) of the fusiform gyrus and the prefrontal cortex in patients with schizophrenia (Silverstein et al., 2010; Turner et al., 2013; Yu et al., 2013; Xu et al., 2015). We speculate that such enhanced local activity in both brain regions might result in more coactivations between them so that their functional connectivity significantly increases in psychosis.

### Altered Features of Dynamic FNC Brain States

During the recent decade, increasing literature has shown that the functional connectivity is highly dynamic even during the resting-state and the dynamic functional connectivity are neuronally original (Hutchison et al., 2013; Allen et al., 2014). It is believed that capturing the functional connectivity on a more detailed temporal scale will provide valuable information underlying the brain mechanisms and disease pathophysiology (Calhoun et al., 2014; Zalesky et al., 2014; Abrol et al., 2017; Fu et al., 2020). In this work, by using a *k*-mean clustering method, three dFNC states were identified reoccurring across windows and individuals. This observation is similar to a previous finding showing that HCs, schizophrenia patients, and individuals with psychotic experiences had similar spatial maps of brain states which were captured by voxel-based functional activity (Wang et al., 2021). Among the three dFNC states, one of them had the largest fractional rate of occurrence and showed diffused connectivity patterns resembling the sFNC (Allen et al., 2014; Yaesoubi et al., 2015; Kim et al., 2017). This state with relatively weak thalamocortical connectivity is typically considered as an average of less variable brain states, associating with reduced vigilance or drowsiness (Allen et al., 2018).

Besides this sparsely connected state, we also observed two strongly connected brain states with significantly positive and negative connectivity between networks, especially a brain state showing obvious antagonism between subcortical and sensory domains and strongly positive connectivity between subcortical and cerebellar domains. Interestingly, both PAP and PnAP groups tended to spend much less time in this state. Considering a wide-range of abnormal cortical-subcortical interactions observed in psychosis (Orr et al., 2014; Woodward and Heckers, 2016) and also in our current static analysis, we speculate that the decreased subcortical involved connectivity may be due to the reduced occurrence in a highly-connected state. That is, the connectivity between subcortical and sensory/cerebellar regions is not consistently disconnected in early psychosis, but spends more time in a weakly connected state and with fewer occurrences in the strongly connected state so that its strength appears reduced in an average sense, thus the disruption is in the transient behavior over time. Similar decreased occurrences to strong dFNC states were identified in psychosis populations, including schizophrenia (Damaraju et al., 2014; Fu et al., 2018), bipolar disorder (Rashid et al., 2014), and major depressive disorder (Zhi et al., 2018). Along with our results, the overall findings indicate that although affective and non-affective psychosis have different neurobiological underpinnings, they share a common thalamocortical dysrhythmia model (Vanneste et al., 2018). We further found that the diminished occurrence in state 2 might cause a re-distribution of the occurrence of the other two brain states, and the re-distribution is associated with the severity of psychotic symptoms. The fractional rate of state 1 is negatively correlated with PANSS while the fractional rate of state 3 is positively correlated with PANSS. We speculate that the patients with early psychosis might re-distribute the occurrence of states 1 and 3. A patient with more severe psychosis symptoms will have more occurrences to state 3 and fewer occurrences to state 1. In contrast, a patient with fewer psychosis symptoms will have more occurrences to state 1 and fewer occurrences to state 3. Our results suggest that the re-distribution of the changed occurrences of the brain states may underlie some core symptoms in psychosis. Except for similarity, PAP group also showed a unique abnormality in dFNC. PAP had a significantly increased number of transitions between states compared with HCs and PnAP, indicating more variable resting-state brain connections in PAP. Previous studies have shown that affective psychosis such as major depressive disorder had significantly increased temporal instability in functional connectivity (Kaiser et al., 2016). One potential interpretation of the unstable connectivity in PAP is that the highly variable connectivity may signify increased sensitivity to salient emotional information and weaknesses in brain circuits responsible for cognitive control and thus can be associated with executive functioning deficits in PAP, such as difficulty inhibiting emotional distraction (Banich et al., 2000; Kaiser et al., 2016).

Previous studies have identified wide-spread abnormalities in multisensory information processing and integration in psychosis (Van Den Heuvel et al., 2010; Cabral et al., 2012; Ajilore et al., 2014). The aberrant brain graphs have also been observed in schizophrenia from the dynamic perspective (Yu et al., 2015; Fu et al., 2021). In this work, we examined the topologic measures in early psychosis transiently within each dFNC state and found significantly disrupted functional integration in brain networks. For both PAP and PnAP groups, the MTG, IOG, precuneus, and cerebellum showed loss of brain efficiency within dFNC state 1, an intermediate state with relatively strong connectivity patterns and high network efficiency. Interestingly, a similar dFNC state with median connectivity strength and network efficiency was identified with reduced local efficiency in schizophrenia (Fu et al., 2021). Our results extend this finding by showing that the loss of transiently local efficiency in both affective and non-affective psychosis, during the initial onset of psychotic symptoms. Our results might also unveil a potential relationship between structural abnormalities and functional abnormalities in early psychosis. There is mounting evidence to support the notion of a reduction in gray matter volume of the temporal lobe, occipital lobe, and cerebellum in different psychosis (Andreasen et al., 1994; Onitsuka et al., 2004; Kuroki et al., 2006; Peng et al., 2011). The reduction of brain volume will limit the BOLD fluctuations within these regions when there is a strong requirement of neural activation during the intermediate state, and the restricted local activities will affect the information processing and integration, reflecting by transiently decreased regional efficiency.

### Limitation and Future Direction

There are some issues and limitations in this study that may deserve investigation in future work. Firstly, although we have observed associations of connectivity metrics with PANSS scores, the correlation values are relatively low, and their p values cannot survive multiple comparison correction. We argue that these relatively weak significances may be due to several reasons. On one hand, it has been shown that psychosis is a heterogeneous condition with a wide range of variations of functional connectivity within psychosis. The heterogeneity of psychosis etiology, behaviors, and cognition might be a possible cause of the weak correlations observed. On the other hand, the current data are collected by multiple sites with unbalance samples across groups. Although we have tried to control for this potential confounding effect by covarying for site, such differences might still influence the properties of the whole dataset and impact the correlation analysis.

Another limitation is that the associations with the overall PANSS score were observed by combining both patient groups. Although we also performed correlation analysis with YMRS and MADRS within each patient group in the Supplementary Materials, the correlations and the significance are relatively weak. In this work, we used an open-source HCP dataset which collected MRI data for two psychosis groups and a control group. However, this dataset included only 34 affective psychosis patients and this number is relatively small. In addition, to guarantee the accuracy of independent component analysis and the FNC estimation, we used very strict criteria for subject selection (Fu et al., 2018, 2019a). This subject selection criteria further resulted in fewer subjects in the PAP group (32 subjects). We consider that such a small sample size might not have enough power for obtaining reliable correlation results. Therefore, we did not perform correlation analysis separately for each patient group in the main text. In future studies with more subjects and balanced sample sizes, we will extend our present findings by performing the correlation analysis for each patient group to examine whether these observed FNC differences reflect distinct clinical profiles of affective and non-affective psychosis.

Although in the present study we have used a fMRI dataset shared by the HCP database which has been collected by a multi-band acquisition technique with relatively high temporal resolution, there are still some participants that did not enter all dFNC states. Conventional resting-state acquisition parameters are challenging for that they might not be optimized for the exploration of dynamic functional states. We believe that a longer duration with higher temporal resolution would be better for validating the dynamic patterns in brain connectivity states in the future.

In this study, we were mainly working on the statistical comparisons between patients and controls and the correlation analysis between connectivity features and symptom scores. We did not focus on the clinical usage such as the classification of diseases, and the evaluation of whether functional connectivity can predict symptoms in early psychosis or the conversion to chronic psychosis. However, our framework can be potentially applied for individual-subject classification (or prediction) in future work by combining it with machine learning models. One possible strategy is to use the leave-one-out or k-folds strategies. That is, the subjects are randomly divided into training and testing groups. For the training group, we can identify connectivity patterns that discriminate between controls and patients and then build a classifier/model using these features. For the testing group, we can measure the distance between the subject-level dFNC estimates and the centroids obtained in the training group and computed the subject-specific state measures. Finally, test (hold out) subjects with connectivity features from both static and dynamic perspectives can be classified by the trained model.

## Conclusion

Previous studies have reported numerous static and dynamic functional connectivity abnormalities in chronic psychosis. In this work, by exploring the functional brain connectivity using static and dynamic FNC, our results revealed that early phase PnAP and PAP groups shared similar and also specific abnormalities in static and dynamic brain characteristics. Additionally, we found that the FNC measures were significantly correlated with the symptom scores. Overall, our findings provide strong evidence that affective and non-affective psychosis show overlapping and unique brain manifestations within 5 years of the initial onset of psychotic symptoms, reflecting by the functional connectivity abnormalities during the resting state. Given that similar abnormalities have been widely observed in chronic psychosis, we propose that the abnormal resting-state connectivity from both static and dynamic perspectives might help to depict a more comprehensive picture of brain mechanisms and disease pathology in subjects along the psychosis continuum.

## Data Availability Statement

Publicly available dataset was analyzed in this study. This data can be found here: https://nda.nih.gov/general-query.html?q=query=featureddatasets:Connectomes%20Related%20to%20Human%20Disease.

## Ethics Statement

The dataset involving human participants was reviewed and approved by The Human Connectome Project. The participants provided their written informed consent to participate in this study.

## Author Contributions

ZF and VC designed the study. ZF and AI analyzed and interpreted the data. ZF and JS wrote the manuscript. All authors revised the manuscript.

## Conflict of Interest

The authors declare that the research was conducted in the absence of any commercial or financial relationships that could be construed as a potential conflict of interest.

## Footnotes

1 http://www.humanconnectomeproject.org/data/

2 https://nda.nih.gov/

3 https://sites.google.com/site/bctnet/

## References

[B1] Abrol A. Damaraju E. Miller R. L. Stephen J. M. Claus E. D. Mayer A. R. (2017). Replicability of time-varying connectivity patterns in large resting state fMRI samples. *Neuroimage* 163 160–176. 10.1016/j.neuroimage.2017.09.020 28916181 PMC5775892

[B2] Ajilore O. Lamar M. Leow A. Zhang A. Yang S. Kumar A. (2014). Graph theory analysis of cortical-subcortical networks in late-life depression. *Am. J. Geriatr. Psychiatry* 22 195–206. 10.1016/j.jagp.2013.03.005 23831171 PMC3858393

[B3] Allen E. A. Damaraju E. Eichele T. Wu L. Calhoun V. D. (2018). EEG signatures of dynamic functional network connectivity states. *Brain Topogr.* 31 101–116. 10.1007/s10548-017-0546-2 28229308 PMC5568463

[B4] Allen E. A. Damaraju E. Plis S. M. Erhardt E. B. Eichele T. Calhoun V. D. (2014). Tracking whole-brain connectivity dynamics in the resting state. *Cereb. Cortex* 24 663–676. 10.1093/cercor/bhs352 23146964 PMC3920766

[B5] Amador X. F. David A. S. (1999). Insight and psychosis. *Nord. J. Psychiatry* 53 467–468.

[B6] American Psychological Association [APA] (2013). *Diagnostic and Statistical Manual of Mental Disorders: Depressive Disorders.* Washington, DC: American Psychiatric Publishing.

[B7] Andreasen N. C. (1997). The role of the thalamus in schizophrenia. *Can. J. Psychiatry* 42 27–33. 10.1177/070674379704200104 9040920

[B8] Andreasen N. C. Flashman L. Flaum M. Arndt S. Swayze V. O’leary D. S. (1994). Regional brain abnormalities in schizophrenia measured with magnetic resonance imaging. *JAMA* 272 1763–1769. 10.1001/jama.1994.03520220057031 7966925

[B9] Arciniegas D. B. (2015). Psychosis. *Contin. Lifelong Learn. Neurol.* 21 715–736. 10.1212/01.CON.0000466662.89908.e7 PMC4455840 26039850

[B10] Baker J. T. Holmes A. J. Masters G. A. Yeo B. T. T. Krienen F. Buckner R. L. (2014). Disruption of cortical association networks in schizophrenia and psychotic bipolar disorder. *JAMA Psychiatry* 71 109–118. 10.1001/jamapsychiatry.2013.3469 24306091 PMC4435541

[B11] Banich M. T. Milham M. P. Atchley R. A. Cohen N. J. Webb A. Wszalek T. (2000). Prefrontal regions play a predominant role in imposing an attentional “set”: evidence from fMRI. *Cogn. Brain Res.* 10 1–9. 10.1016/s0926-6410(00)00015-x 10978687

[B12] Benjamini Y. Hochberg Y. (1995). Controlling the false discovery rate: a practical and powerful approach to multiple testing. *J. R. Stat. Soc. Ser. B* 57 289–300. 10.1111/j.2517-6161.1995.tb02031.x

[B13] Bergé D. Lesh T. A. Smucny J. Carter C. S. (2020). Improvement in prefrontal thalamic connectivity during the early course of the illness in recent-onset psychosis: a 12-month longitudinal follow-up resting-state fMRI study. *Psychol. Med.* 16 1–9. 10.1017/s0033291720004808 33323140 PMC9307321

[B14] Bernard J. A. Dean D. J. Kent J. S. Orr J. M. Pelletier-Baldelli A. Lunsford-Avery J. R. (2014). Cerebellar networks in individuals at ultra high-risk of psychosis: impact on postural sway and symptom severity. *Hum. Brain Mapp.* 35 4064–4078. 10.1002/hbm.22458 24464473 PMC4107098

[B15] Bird V. Premkumar P. Kendall T. Whittington C. Mitchell J. Kuipers E. (2010). Early intervention services, cognitive-behavioural therapy and family intervention in early psychosis: systematic review. *Br. J. Psychiatry* 197 350–356. 10.1192/bjp.bp.109.074526 21037211 PMC2966501

[B16] Byne W. Hazlett E. A. Buchsbaum M. S. Kemether E. (2009). The thalamus and schizophrenia: current status of research. *Acta Neuropathol.* 117 347–368. 10.1007/s00401-008-0404-0 18604544

[B17] Cabral J. Kringelbach M. L. Deco G. (2012). Functional graph alterations in schizophrenia: a result from a global anatomic decoupling? *Pharmacopsychiatry* 45(Suppl. 1), 57–64. 22565236 10.1055/s-0032-1309001

[B18] Calhoun V. D. Miller R. Pearlson G. Adali T. (2014). The chronnectome: time-varying connectivity networks as the next frontier in fMRI data discovery. *Neuron* 84 262–274. 10.1016/j.neuron.2014.10.015 25374354 PMC4372723

[B19] Cannon T. D. (2015). How schizophrenia develops: cognitive and brain mechanisms underlying onset of psychosis. *Trends Cogn. Sci.* 19 744–756. 10.1016/j.tics.2015.09.009 26493362 PMC4673025

[B20] Canuet L. Ishii R. Pascual-Marqui R. D. Iwase M. Kurimoto R. Aoki Y. (2011). Resting-state EEG source localization and functional connectivity in schizophrenia-like psychosis of epilepsy. *PLoS One* 6:e0027863. 10.1371/journal.pone.0027863 PMC3220705 22125634

[B21] Carhart-Harris R. L. Leech R. Erritzoe D. Williams T. M. Stone J. M. Evans J. (2013). Functional connectivity measures after psilocybin inform a novel hypothesis of early psychosis. *Schizophr. Bull.* 39 1343–1351. 10.1093/schbul/sbs117 23044373 PMC3796071

[B22] Damaraju E. Allen E. A. Belger A. Ford J. M. McEwen S. Mathalon D. H. (2014). Dynamic functional connectivity analysis reveals transient states of dysconnectivity in schizophrenia. *NeuroImage Clin.* 5 298–308. 10.1016/j.nicl.2014.07.003 25161896 PMC4141977

[B23] Dandash O. Fornito A. Lee J. Keefe R. S. E. Chee M. W. L. Adcock R. A. (2014). Altered striatal functional connectivity in subjects with an at-risk mental state for psychosis. *Schizophr. Bull.* 40 904–913. 10.1093/schbul/sbt093 23861539 PMC4059431

[B24] Du Y. Fu Z. Sui J. Gao S. Xing Y. Lin D. (2020). NeuroMark: an automated and adaptive ICA based pipeline to identify reproducible fMRI markers of brain disorders. *NeuroImage Clin.* 28:102375. 10.1016/j.nicl.2020.102375 32961402 PMC7509081

[B25] Friston K. J. (1998). The disconnection hypothesis. *Schizophr. Res.* 176 115–125. 10.1016/s0920-9964(97)00140-0 9549774

[B26] Friston K. J. (1999). Schizophrenia and the disconnection hypothesis. *Acta Psychiatr. Scand. Suppl.* 395 68–79. 10.1111/j.1600-0447.1999.tb05985.x 10225335

[B27] Fu Z. Caprihan A. Chen J. Du Y. Adair J. C. Sui J. (2019a). Altered static and dynamic functional network connectivity in Alzheimer’s disease and subcortical ischemic vascular disease: shared and specific brain connectivity abnormalities. *Hum. Brain Mapp.* 40 3203–3221. 10.1002/hbm.24591 30950567 PMC6865624

[B28] Fu Z. Iraji A. Turner J. A. Sui J. Miller R. Pearlson G. D. (2021). Dynamic state with covarying brain activity-connectivity: on the pathophysiology of schizophrenia. *Neuroimage* 224:117385. 10.1016/j.neuroimage.2020.117385 32950691 PMC7781150

[B29] Fu Z. Sui J. Turner J. A. Du Y. Assaf M. Pearlson G. D. (2020). Dynamic functional network reconfiguration underlying the pathophysiology of schizophrenia and autism spectrum disorder. *Hum. Brain Mapp.* 42 80–94. 10.1002/hbm.25205 32965740 PMC7721229

[B30] Fu Z. Tu Y. Di X. Du Y. Pearlson G. D. Turner J. A. (2018). Characterizing dynamic amplitude of low-frequency fluctuation and its relationship with dynamic functional connectivity: an application to schizophrenia. *Neuroimage* 180 619–631. 10.1016/j.neuroimage.2017.09.035 28939432 PMC5860934

[B31] Fu Z. Tu Y. Di X. Du Y. Sui J. Biswal B. B. (2019b). Transient increased thalamic-sensory connectivity and decreased whole-brain dynamism in autism. *Neuroimage* 190 191–204. 10.1016/j.neuroimage.2018.06.003 29883735 PMC6281849

[B32] Gardner D. M. Murphy A. L. O’Donnell H. Centorrino F. Baldessarini R. J. (2010). International consensus study of antipsychotic dosing. *Am. J. Psychiatry* 167 686–693. 10.1176/appi.ajp.2009.09060802 20360319

[B33] Harrigan S. M. McGorry P. D. Krstev H. (2003). Does treatment delay in first-episode psychosis really matter? *Psychol. Med.* 33 97–110. 10.1017/s003329170200675x 12537041

[B34] Hazlett E. A. Buchsbaum M. S. Byne W. Wei T. C. Spiegel-Cohen J. Geneve C. (1999). Three-dimensional analysis with MRI and PET of the size, shape, and function of the thalamus in the schizophrenia spectrum. *Am. J. Psychiatry* 156 1190–1199. 10450259 10.1176/ajp.156.8.1190

[B35] Hutchison R. M. Womelsdorf T. Allen E. A. Bandettini P. A. Calhoun V. D. Corbetta M. (2013). Dynamic functional connectivity: promise, issues, and interpretations. *Neuroimage* 80 360–378. 10.1016/j.neuroimage.2013.05.079 23707587 PMC3807588

[B36] Iraji A. Faghiri A. Fu Z. Rachakonda S. Kochunov P. Belger A. (2021). Multi-spatial scale dynamic interactions between functional sources reveal sex-specific changes in schizophrenia. *bioRxiv [Preprint]* 10.1101/2021.01.04.425222 PMC9208002 35733435

[B37] Iraji A. Fu Z. Damaraju E. DeRamus T. P. Lewis N. Bustillo J. R. (2019). Spatial dynamics within and between brain functional domains: a hierarchical approach to study time-varying brain function. *Hum. Brain Mapp.* 40 1969–1986. 10.1002/hbm.24505 30588687 PMC6692083

[B38] Javitt D. C. (2009). Sensory processing in schizophrenia: neither simple nor intact. *Schizophr. Bull.* 35 1059–1064. 10.1093/schbul/sbp110 19833806 PMC2762632

[B39] Javitt D. C. Freedman R. (2015). Sensory processing dysfunction in the personal experience and neuronal machinery of schizophrenia. *Am. J. Psychiatry* 172 17–31. 10.1176/appi.ajp.2014.13121691 25553496 PMC4501403

[B40] Kaiser R. H. Whitfield-Gabrieli S. Dillon D. G. Goer F. Beltzer M. Minkel J. (2016). Dynamic resting-state functional connectivity in major depression. *Neuropsychopharmacology* 41 1822–1830. 26632990 10.1038/npp.2015.352 PMC4869051

[B41] Kaufmann T. Skåtun K. C. Alnæs D. Doan N. T. Duff E. P. Tønnesen S. (2015). Disintegration of sensorimotor brain networks in schizophrenia. *Schizophr. Bull.* 41 1326–1335. 10.1093/schbul/sbv060 25943122 PMC4601711

[B42] Kay S. R. Fiszbein A. Opler L. A. (1987). The positive and negative syndrome scale (PANSS) for schizophrenia. *Schizophr. Bull.* 13 261–276. 10.1093/schbul/13.2.261 3616518

[B43] Keshavan M. S. Giedd J. Lau J. Y. F. Lewis D. A. Paus T. (2014). Changes in the adolescent brain and the pathophysiology of psychotic disorders. *Lancet Psychiatry* 1 549–558. 10.1016/s2215-0366(14)00081-9 26361314

[B44] Khadka S. Meda S. A. Stevens M. C. Glahn D. C. Calhoun V. D. Sweeney J. A. (2013). Is aberrant functional connectivity a psychosis endophenotype? a resting state functional magnetic resonance imaging study. *Biol. Psychiatry* 74 458–466. 10.1016/j.biopsych.2013.04.024 23746539 PMC3752322

[B45] Kim J. Criaud M. Cho S. S. Díez-Cirarda M. Mihaescu A. Coakeley S. (2017). Abnormal intrinsic brain functional network dynamics in Parkinson’s disease. *Brain* 140 2955–2967. 10.1093/brain/awx233 29053835 PMC5841202

[B46] Krukow P. Jonak K. Karakuła-Juchnowicz H. Podkowiński A. Jonak K. Borys M. (2018). Disturbed functional connectivity within the left prefrontal cortex and sensorimotor areas predicts impaired cognitive speed in patients with first-episode schizophrenia. *Psychiatry Res. Neuroimaging* 275 28–35. 10.1016/j.pscychresns.2018.03.001 29526598

[B47] Kuroki N. Shenton M. E. Salisbury D. F. Hirayasu Y. Onitsuka T. Ersner-Hershfield H. (2006). Middle and inferior temporal gyrus gray matter volume abnormalities in first-episode schizophrenia: an MRI Study. *Am. J. Psychiatry* 163 2103–2110. 10.1176/ajp.2006.163.12.2103 17151161 PMC2766919

[B48] Leiner H. C. Leiner A. L. Dow R. S. (1993). Cognitive and language functions of the human cerebellum. *Trends Neurosci.* 16 444–447. 10.1016/0166-2236(93)90072-t 7507614

[B49] Li K. Fu Z. Luo X. Zeng Q. Huang P. Zhang M.-M. (2020). The influence of cerebral small vessel disease on static and dynamic functional network connectivity in subjects along Alzheimer’s disease continuum. *Brain Connect.* 11 189–200. 10.1089/brain.2020.0819 33198482 PMC8080908

[B50] Lurie D. J. Kessler D. Bassett D. S. Betzel R. F. Breakspear M. Kheilholz S. (2020). Questions and controversies in the study of time-varying functional connectivity in resting fMRI. *Netw. Neurosci.* 4 30–69. 32043043 10.1162/netn_a_00116 PMC7006871

[B51] Mallikarjun P. K. Lalousis P. A. Dunne T. F. Heinze K. Reniers R. L. Broome M. R. (2018). Aberrant salience network functional connectivity in auditory verbal hallucinations: a first episode psychosis sample. *Transl. Psychiatry* 8:69. 10.1038/s41398-018-0118-6 PMC5913255 29581420

[B52] Mamah D. Barch D. M. Repovš G. (2013). Resting state functional connectivity of five neural networks in bipolar disorder and schizophrenia. *J. Affect. Disord.* 150 601–609. 10.1016/j.jad.2013.01.051 23489402 PMC3749249

[B53] Meda S. A. Gill A. Stevens M. C. Lorenzoni R. P. Glahn D. C. Calhoun V. D. (2012). Differences in resting-state functional magnetic resonance imaging functional network connectivity between schizophrenia and psychotic bipolar probands and their unaffected first-degree relatives. *Biol. Psychiatry* 71 881–889. 10.1016/j.biopsych.2012.01.025 22401986 PMC3968680

[B54] Mennigen E. Fryer S. L. Rashid B. Damaraju E. Du Y. Loewy R. L. (2019). Transient patterns of functional dysconnectivity in clinical high risk and early illness schizophrenia individuals compared with healthy controls. *Brain Connect.* 9 60–76. 10.1089/brain.2018.0579 29855202 PMC6390658

[B55] Mennigen E. Miller R. L. Rashid B. Fryer S. L. Loewy R. L. Stuart B. K. (2018). Reduced higher-dimensional resting state fMRI dynamism in clinical high-risk individuals for schizophrenia identified by meta-state analysis. *Schizophr. Res.* 201 217–223. 10.1016/j.schres.2018.06.007 29907493 PMC6252113

[B56] Montgomery S. A. Asberg M. (1979). A new depression scale designed to be sensitive to change. *Br. J. Psychiatry* 134 382–389. 10.1192/bjp.134.4.382 444788

[B57] Onitsuka T. Shenton M. E. Salisbury D. F. Dickey C. C. Kasai K. Toner S. K. (2004). Middle and inferior temporal gyrus gray matter volume abnormalities in chronic schizophrenia: an MRI study. *Am. J. Psychiatry* 161 1603–1611. 10.1176/appi.ajp.161.9.1603 15337650 PMC2793337

[B58] Orr J. M. Turner J. A. Mittal V. A. (2014). Widespread brain dysconnectivity associated with psychotic-like experiences in the general population. *NeuroImage Clin.* 4 343–351. 10.1016/j.nicl.2014.01.006 24501703 PMC3913833

[B59] Palaniyappan L. Liddle P. F. (2014). Diagnostic discontinuity in psychosis: a combined study of cortical gyrification and functional connectivity. *Schizophr. Bull.* 40 675–684. 10.1093/schbul/sbt050 23615812 PMC3984507

[B60] Pavuluri M. N. Passarotti A. (2008). Neural bases of emotional processing in pediatric bipolar disorder. *Expert Rev. Neurother.* 8 1381–1387. 10.1586/14737175.8.9.1381 18759550

[B61] Pavuluri M. N. O’Connor M. M. Harral E. Sweeney J. A. (2007). Affective neural circuitry during facial emotion processing in pediatric bipolar disorder. *Biol. Psychiatry* 62 158–167. 10.1016/j.biopsych.2006.07.011 17097071

[B62] Peng J. Liu J. Nie B. Li Y. Shan B. Wang G. (2011). Cerebral and cerebellar gray matter reduction in first-episode patients with major depressive disorder: a voxel-based morphometry study. *Eur. J. Radiol.* 80 395–399. 10.1016/j.ejrad.2010.04.006 20466498

[B63] Rashid B. Damaraju E. Pearlson G. D. Calhoun V. D. (2014). Dynamic connectivity states estimated from resting fMRI Identify differences among Schizophrenia, bipolar disorder, and healthy control subjects. *Front. Hum. Neurosci.* 8:897. 10.3389/fnhum.2014.00897 PMC4224100 25426048

[B64] Satterthwaite T. D. Baker J. T. (2015). How can studies of resting-state functional connectivity help us understand psychosis as a disorder of brain development? *Curr. Opin. Neurobiol.* 30 85–91. 10.1016/j.conb.2014.10.005 25464373 PMC4293321

[B65] Schutter D. J. L. G. van Honk J. (2006). An electrophysiological link between the cerebellum, cognition and emotion: frontal theta EEG activity to single-pulse cerebellar TMS. *Neuroimage* 33 1227–1231. 10.1016/j.neuroimage.2006.06.055 17023183

[B66] Shinn A. K. Roh Y. S. Ravichandran C. T. Baker J. T. Öngür D. Cohen B. M. (2017). Aberrant cerebellar connectivity in bipolar disorder with psychosis. *Biol. Psychiatry Cogn. Neurosci. Neuroimaging* 2 438–448. 10.1016/j.bpsc.2016.07.002 28730183 PMC5512437

[B67] Silverstein S. M. All S. D. Kasi R. Berten S. Essex B. Lathrop K. L. (2010). Increased fusiform area activation in schizophrenia during processing of spatial frequency-degraded faces, as revealed by fMRI. *Psychol. Med.* 40 1159–1169. 10.1017/s0033291709991735 19895721

[B68] Tu Y. Fu Z. Mao C. Falahpour M. Gollub R. L. Park J. (2020). Distinct thalamocortical network dynamics are associated with the pathophysiology of chronic low back pain. *Nat. Commun.* 11:3948. 10.1038/s41467-020-17788-z PMC7414843 32769984

[B69] Turner J. A. Damaraju E. Van Erp T. G. M. Mathalon D. H. Ford J. M. Voyvodic J. (2013). A multi-site resting state fMRI study on the amplitude of low frequency fluctuations in schizophrenia. *Front. Neurosci.* 7:137. 10.3389/fnins.2013.00137 PMC3737471 23964193

[B70] Van Den Heuvel M. P. Mandl R. C. W. Stam C. J. Kahn R. S. Hulshoff Pol H. E. (2010). Aberrant frontal and temporal complex network structure in schizophrenia: a graph theoretical analysis. *J. Neurosci.* 30 15915–15926. 10.1523/jneurosci.2874-10.2010 21106830 PMC6633761

[B71] Vanneste S. Song J. J. De Ridder D. (2018). Thalamocortical dysrhythmia detected by machine learning. *Nat. Commun.* 9:1103. 10.1038/s41467-018-02820-0 PMC5856824 29549239

[B72] Wang D. Li M. Wang M. Schoeppe F. Ren J. Chen H. (2020). Individual-specific functional connectivity markers track dimensional and categorical features of psychotic illness. *Mol. Psychiatry* 25 2119–2129. 10.1038/s41380-018-0276-1 30443042 PMC6520219

[B73] Wang D. Peng X. Pelletier-Baldelli A. Orlov N. Farabaugh A. Nasr S. (2021). Altered temporal, but intact spatial, features of transient network dynamics in psychosis. *Mol. Psychiatry.* 10.1038/s41380-020-00983-1 PMC8286268 33462330

[B74] Woodward N. D. Heckers S. (2016). Mapping thalamocortical functional connectivity in chronic and early stages of psychotic disorders. *Biol. Psychiatry* 79 1016–1025. 10.1016/j.biopsych.2015.06.026 26248537 PMC4698230

[B75] World Health Organization (2001). The world health report 2001 — mental health: new understanding, new hope. *Bull. World Health Organ.* 79 1085–1085.

[B76] Xu Y. Zhuo C. Qin W. Zhu J. Yu C. (2015). Altered spontaneous brain activity in schizophrenia: a meta-analysis and a large-sample study. *Biomed Res. Int.* 2015 1–11. 10.1155/2015/204628 26180786 PMC4477065

[B77] Yaesoubi M. Allen E. A. Miller R. L. Calhoun V. D. (2015). Dynamic coherence analysis of resting fMRI data to jointly capture state-based phase, frequency, and time-domain information. *Neuroimage* 120 133–142. 10.1016/j.neuroimage.2015.07.002 26162552 PMC4589498

[B78] Young R. C. Biggs J. T. Ziegler V. E. Meyer D. A. (1978). A rating scale for mania: reliability, validity and sensitivity. *Br. J. Psychiatry* 133 429–435. 10.1192/bjp.133.5.429 728692

[B79] Yu Q. Erhardt E. B. Sui J. Du Y. He H. Hjelm D. (2015). Assessing dynamic brain graphs of time-varying connectivity in fMRI data: application to healthy controls and patients with schizophrenia. *Neuroimage* 107 345–355. 10.1016/j.neuroimage.2014.12.020 25514514 PMC4300250

[B80] Yu R. Hsieh M. H. Wang H. L. S. Liu C. M. Liu C. C. Hwang T. J. (2013). Frequency dependent alterations in regional homogeneity of baseline brain activity in schizophrenia. *PLoS One* 8:e0057516. 10.1371/journal.pone.0057516 PMC3590274 23483911

[B81] Zalesky A. Fornito A. Cocchi L. Gollo L. L. Breakspear M. (2014). Time-resolved resting-state brain networks. *Proc. Natl. Acad. Sci. U. S. A.* 111 10341–10346. 24982140 10.1073/pnas.1400181111 PMC4104861

[B82] Zhang M. Yang F. Fan F. Wang Z. Hong X. Tan Y. (2020). Abnormal amygdala subregional-sensorimotor connectivity correlates with positive symptom in schizophrenia. *NeuroImage Clin.* 26:102218. 10.1016/j.nicl.2020.102218 32126520 PMC7052514

[B83] Zhi D. Calhoun V. D. Lv L. Ma X. Ke Q. Fu Z. (2018). Aberrant dynamic functional network connectivity and graph properties in major depressive disorder. *Front. Psychiatry* 9:339. 10.3389/fpsyt.2018.00339 PMC6080590 30108526

