# Supplementary material for: Whole-Brain Functional Network Connectivity Abnormalities in Affective and Non-Affective Early Phase Psychosis
Source: Front Neurosci. 2021 Jun 18;15:682110. doi: 10.3389/fnins.2021.682110 (PMC8250435; doi:10.3389/fnins.2021.682110)
Supplement: Supplementary file 1 [file Data_Sheet_1.docx]

Supplementary Materials for “Whole-brain Functional Network Connectivity Abnormalities

in Affective and Non-affective Early Phase Psychosis”

# Subject Selection by Comparing Group Mask with Individual Mask

The independent component analysis (ICA) requires that the functional magnetic resonance imaging (fMRI) data have good normalization to the template space. We adopted a method which has been widely applied in our previous studies for subject selection. First, we calculated the individual mask using the first fMRI time volume for each subject by setting voxels which are greater than 90% of the whole brain mean to 1. Next, we computed a group mask by setting voxels which are included in more than 90% of the subjects to 1. After obtaining the individual masks and group mask, we then calculated the spatial correlations between the group mask and each individual mask. The spatial correlations were calculated using voxels within the top 10 slices of the mask, within the bottom 10 slices of the mask and within the whole mask, resulting in three correlations for each subject. If the correlation of a subject is larger than 0.75 for the top 10 slices, larger than 0.55 for the bottom 10 slices, and larger than 0.8 for the whole mask, we included this subject for further analysis.

# Neuromark Framework

The Neuromark used a set of robust network priors to extract comparable ICNs for the HCP_EP dataset. These network priors were extracted via a unified ICA framework. Two healthy controls datasets, human connectome project (HCP, 823 subjects after the subject selection) and genomics superstruct project (GSP, 1005 subjects after the subject selection) were used for the identification of the priors. Group ICA with model order as 100 was performed on the GSP and HCP datasets respectively, and the captured ICs from the two datasets were then matched by comparing their group-level spatial maps. The pairs are considered as consistent and reproducible across GSP and HCP datasets if their spatial correlation ≥ 0.4. Previous studies have shown that a correlation value ≥ 0.25 has been shown to represent a significant correspondence (p < 0.005, corrected) between components. After the matching procedure, the reproducible ICs pairs were further evaluated by 5 neuroimaging experts, who examined the ICs’ peak activations and low-frequency fluctuations of their corresponding time-courses. 53 pairs of ICs were identified as ICNs, arranging into 7 functional domains based on their anatomic and functional prior knowledge. The less noisy ICNs captured from the GSP dataset were used (Note that there are 53 ICNs from HCP which have similar spatial patterns) as the spatial network priors to back-reconstruct spatial maps and time-courses for the HCP_EP data.

# Peak Coordinates and Spatial Maps for Intrinsic Connectivity Networks (ICNs)

Peak Coordinates of Intrinsic Connectivity Networks (ICNs)

| **ICNs** | **X** | **Y** | **Z** |
| --- | --- | --- | --- |
| **Sub-cortical domain (SC)** | | | |
| Caudate (69) | 6.5 | 10.5 | 5.5 |
| Subthalamus/hypothalamus (53) | -2.5 | -13.5 | -1.5 |
| Putamen (98) | -26.5 | 1.5 | -0.5 |
| Caudate (99) | 21.5 | 10.5 | -3.5 |
| Thalamus (45) | -12.5 | -18.5 | 11.5 |
| **Auditory domain (AUD)** | | | |
| Superior temporal gyrus ([STG], 21) | 62.5 | -22.5 | 7.5 |
| Middle temporal gyrus ([MTG], 56) | -42.5 | -6.5 | 10.5 |
| **Sensorimotor domain (SM)** | | | |
| Postcentral gyrus ([PoCG], 3) | 56.5 | -4.5 | 28.5 |
| Left postcentral gyrus ([L PoCG], 9) | -38.5 | -22.5 | 56.5 |
| Paracentral lobule ([ParaCL], 2) | 0.5 | -22.5 | 65.5 |
| Right postcentral gyrus ([R PoCG], 11) | 38.5 | -19.5 | 55.5 |
| Superior parietal lobule ([SPL], 27) | -18.5 | -43.5 | 65.5 |
| Paracentral lobule ([ParaCL], 54) | -18.5 | -9.5 | 56.5 |
| Precentral gyrus ([PreCG], 66) | -42.5 | -7.5 | 46.5 |
| Superior parietal lobule ([SPL], 80) | 20.5 | -63.5 | 58.5 |
| Postcentral gyrus ([PoCG], 72) | -47.5 | -27.5 | 43.5 |
| **Visual domain (VS)** | | | |
| Calcarine gyrus ([CalcarineG], 16) | -12.5 | -66.5 | 8.5 |
| Middle occipital gyrus ([MOG], 5) | -23.5 | -93.5 | -0.5 |
| Middle temporal gyrus ([MTG], 62) | 48.5 | -60.5 | 10.5 |
| Cuneus (15) | 15.5 | -91.5 | 22.5 |
| Right middle occipital gyrus ([R MOG], 12) | 38.5 | -73.5 | 6.5 |
| Fusiform gyrus (93) | 29.5 | -42.5 | -12.5 |
| Inferior occipital gyrus ([IOG], 20) | -36.5 | -76.5 | -4.5 |
| Lingual gyrus ([LingualG], 8) | -8.5 | -81.5 | -4.5 |
| Middle temporal gyrus ([MTG], 77) | -44.5 | -57.5 | -7.5 |
| **Cognitive-control domain (CC)** | | | |
| Inferior parietal lobule ([IPL], 68) | 45.5 | -61.5 | 43.5 |
| Insula (33) | -30.5 | 22.5 | -3.5 |
| Superior medial frontal gyrus ([SMFG], 43) | -0.5 | 50.5 | 29.5 |
| Inferior frontal gyrus ([IFG], 70) | -48.5 | 34.5 | -0.5 |
| Right inferior frontal gyrus ([R IFG], 61) | 53.5 | 22.5 | 13.5 |
| Middle frontal gyrus ([MiFG], 55) | -41.5 | 19.5 | 26.5 |
| Inferior parietal lobule ([IPL], 63) | -53.5 | -49.5 | 43.5 |
| Left inferior parietal lobue ([R IPL], 79) | 44.5 | -34.5 | 46.5 |
| Supplementary motor area ([SMA], 84) | -6.5 | 13.5 | 64.5 |
| Superior frontal gyrus ([SFG], 96) | -24.5 | 26.5 | 49.5 |
| Middle frontal gyrus ([MiFG], 88) | 30.5 | 41.5 | 28.5 |
| Hippocampus ([HiPP], 48) | 23.5 | -9.5 | -16.5 |
| Left inferior parietal lobue ([L IPL], 81) | 45.5 | -61.5 | 43.5 |
| Middle cingulate cortex ([MCC], 37) | -15.5 | 20.5 | 37.5 |
| Inferior frontal gyrus ([IFG], 67) | 39.5 | 44.5 | -0.5 |
| Middle frontal gyrus ([MiFG], 38) | -26.5 | 47.5 | 5.5 |
| Hippocampus ([HiPP], 83) | -24.5 | -36.5 | 1.5 |
| **Default-mode domain (DM)** | | | |
| Precuneus (32) | -8.5 | -66.5 | 35.5 |
| Precuneus (40) | -12.5 | -54.5 | 14.5 |
| Anterior cingulate cortex ([ACC], 23) | -2.5 | 35.5 | 2.5 |
| Posterior cingulate cortex ([PCC], 71) | -5.5 | -28.5 | 26.5 |
| Anterior cingulate cortex ([ACC], 17) | -9.5 | 46.5 | -10.5 |
| Precuneus (51) | -0.5 | -48.5 | 49.5 |
| Posterior cingulate cortex ([PCC], 94) | -2.5 | 54.5 | 31.5 |
| **Cerebellar domain (CB)** | | | |
| Cerebellum ([CB], 13) | -30.5 | -54.5 | -42.5 |
| Cerebellum ([CB], 18) | -32.5 | -79.5 | -37.5 |
| Cerebellum ([CB], 4) | 20.5 | -48.5 | -40.5 |
| Cerebellum ([CB], 7) | 30.5 | -63.5 | -40.5 |


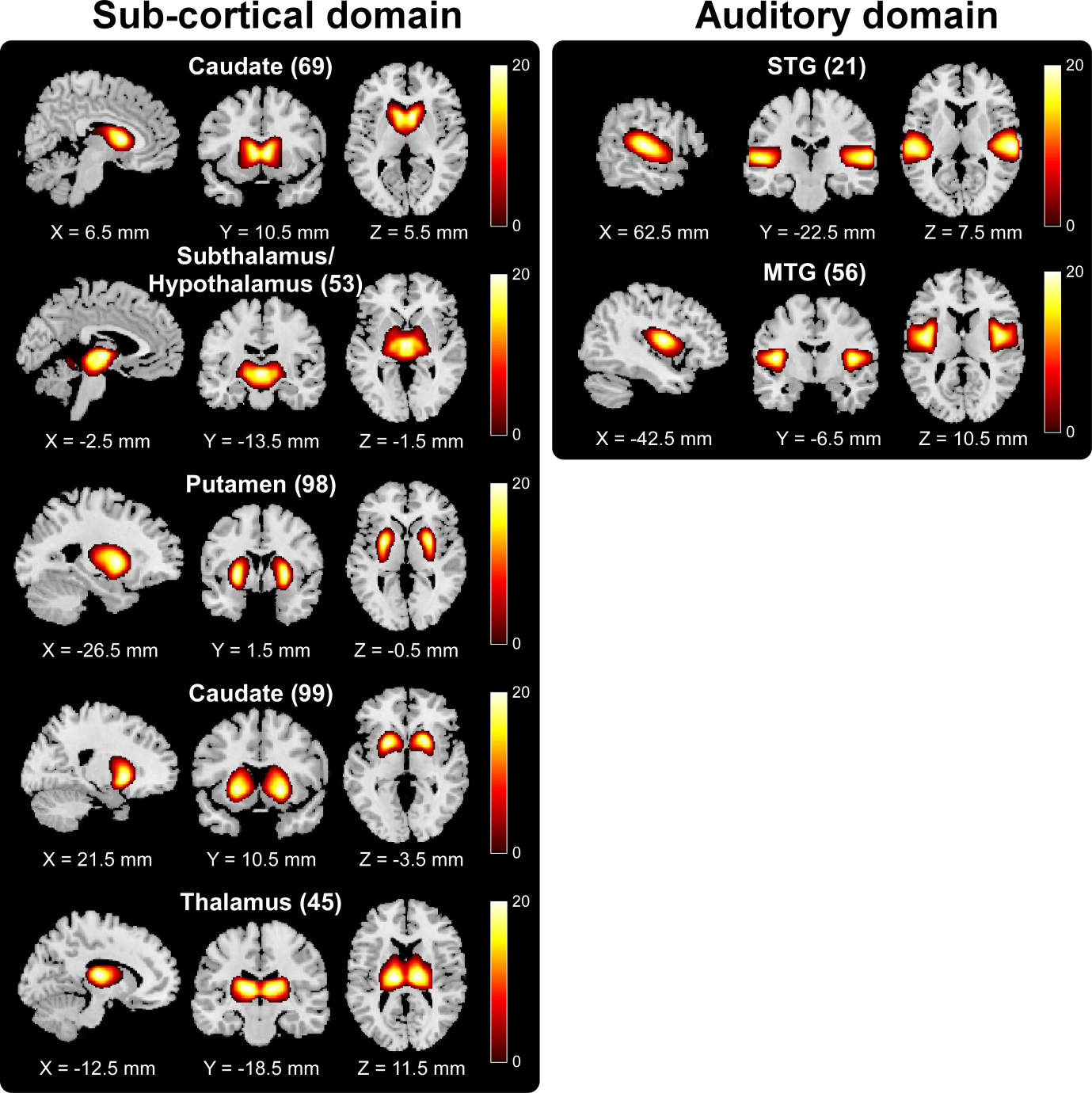


**Spatial maps of intrinsic connectivity networks (ICNs).** ICNs are assigned into seven functional domains, thresholded at |*t*|>10, where one-sample t-statistics have been computed across the single-subject spatial maps.


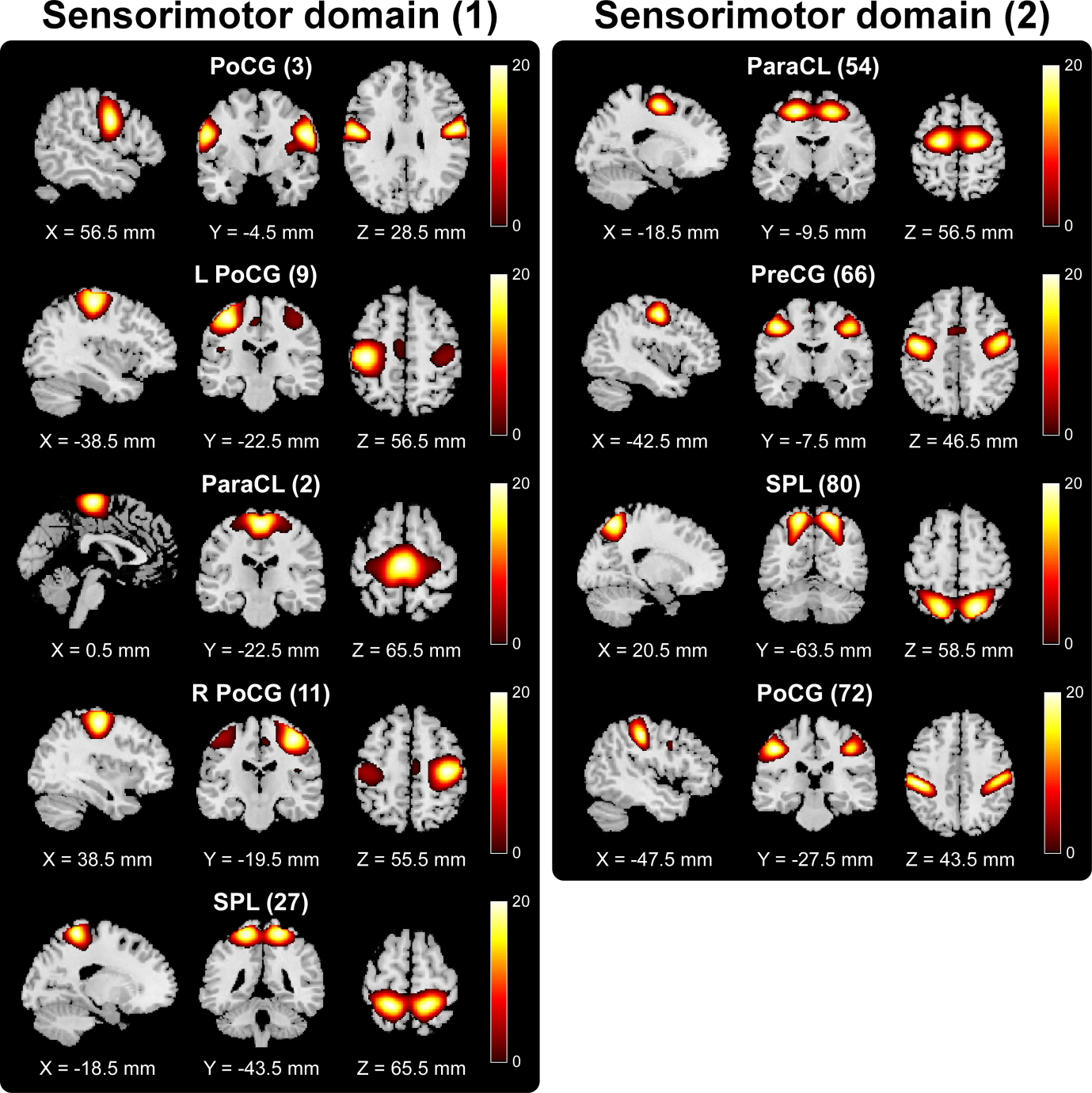


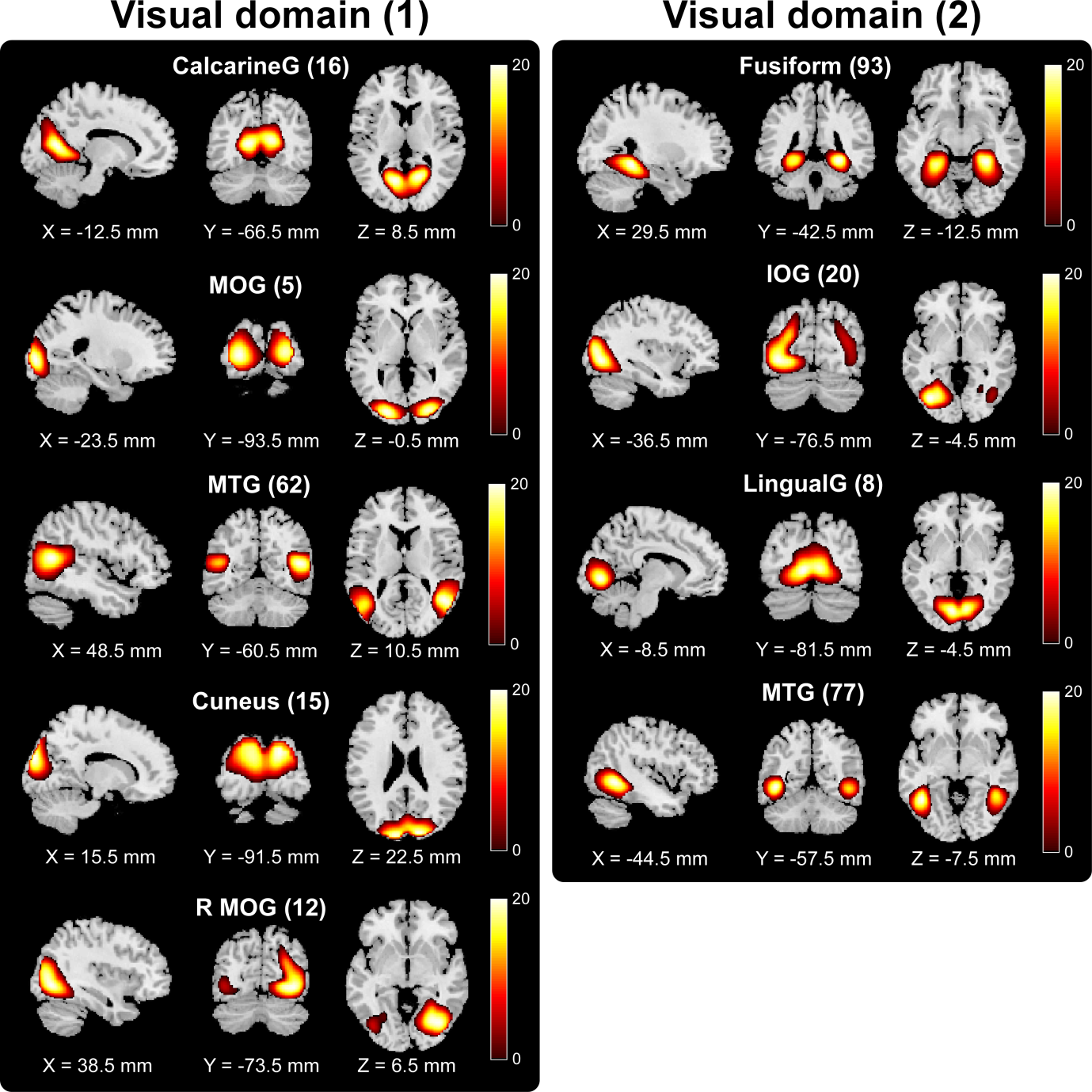


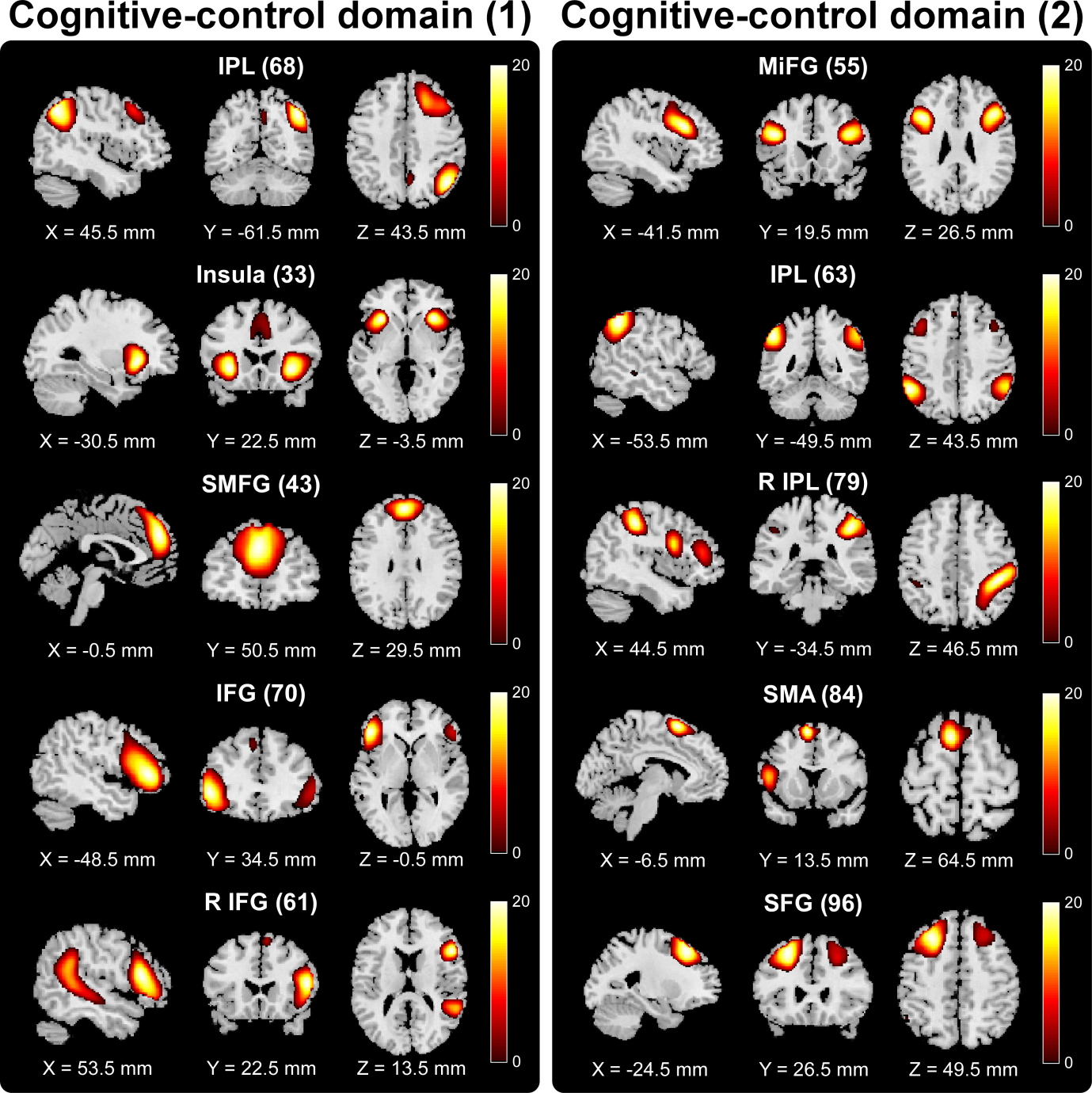


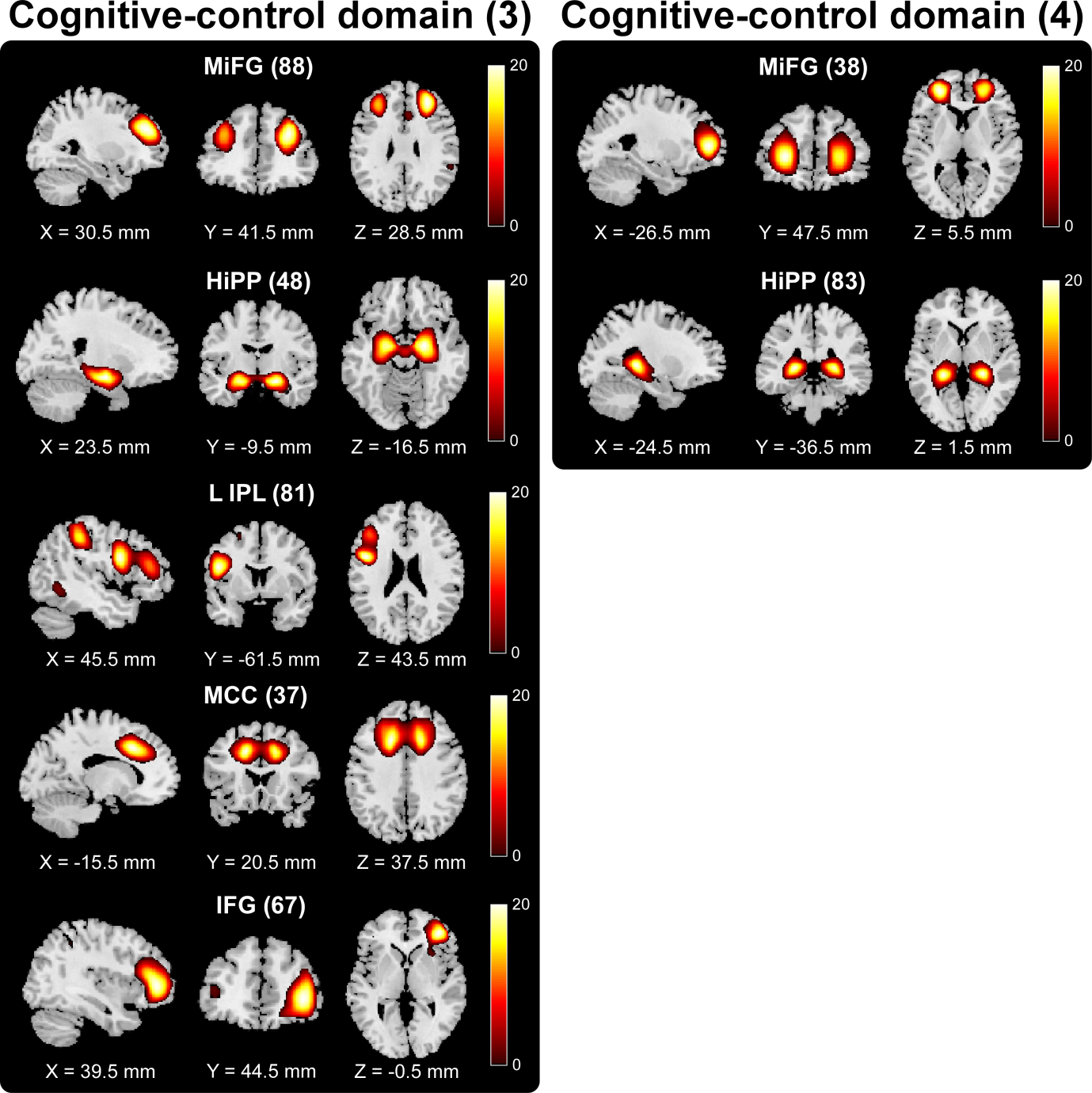


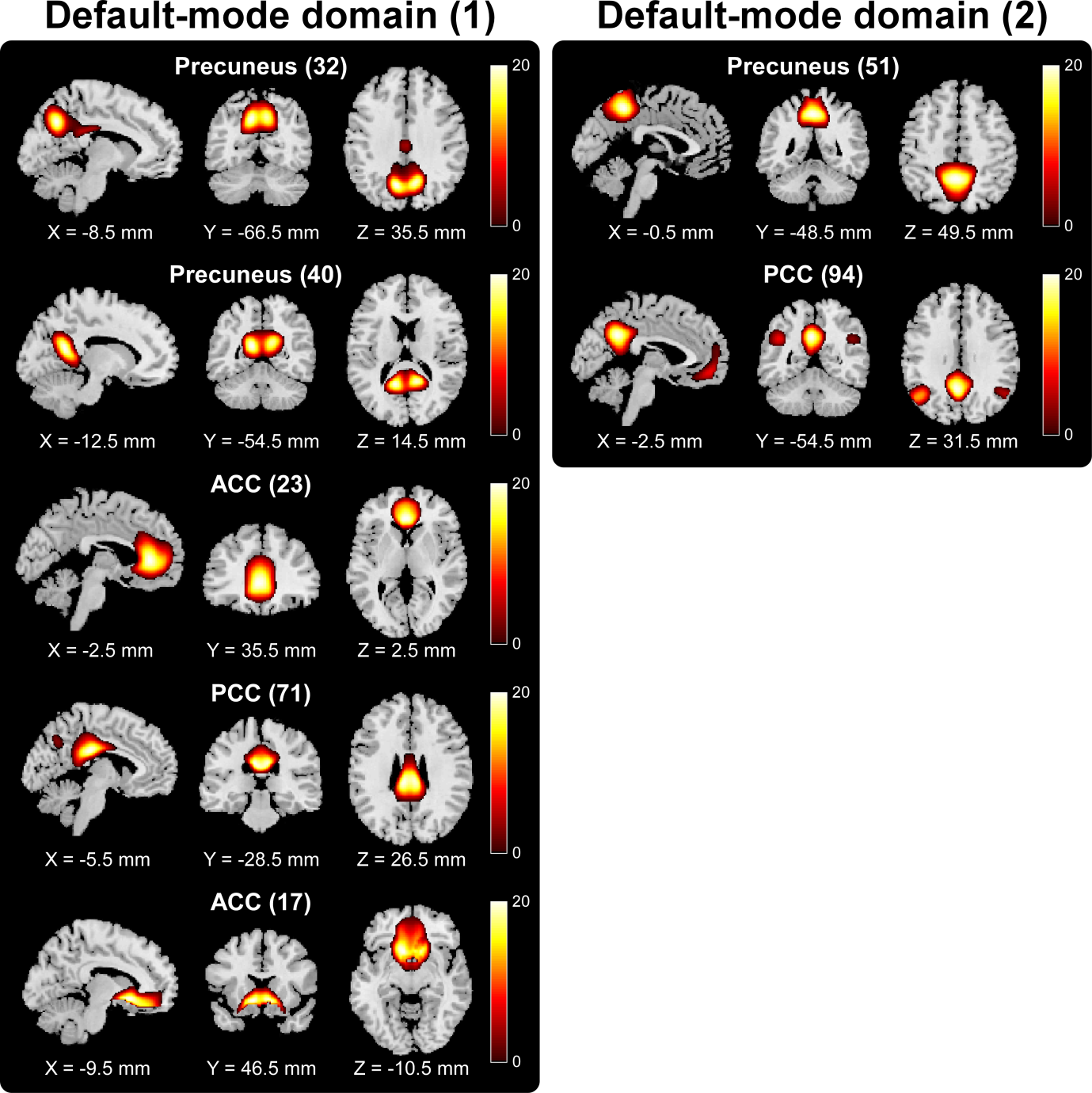


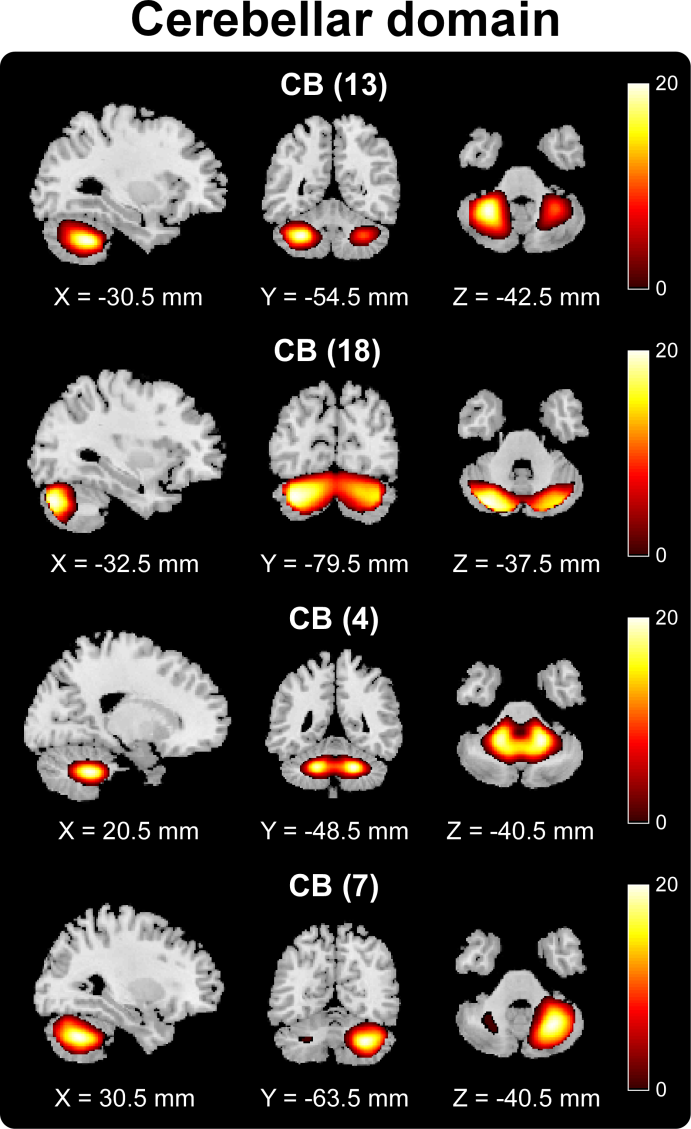


# Replication of Findings by Adding Full IQ as one Covariate

This HCP-EP dataset recorded lifetime antipsychotic medication dosage as CPZ equivalents using the Gardner approach (Gardner et al., 2010). Only 3 patients with affective psychosis and 26 patients with non-affective psychosis after subject selection have CPZ equivalents recorded. There is no significant difference between patient groups (PAP:366.67 ± 208.17; PnAP: 423.08 ± 229.88; p = 0.6886). We extracted the full IQ according to the WASI-II to indicate the overall assessment of cognitive performance. 157 subjects have the WASI-II recorded after the subject selection. Although HCs showed significant higher IQ (115.96 ± 10.78) than both PAP (106.61 ± 14.80; p = 0.0018) and PnAP (98.70 ± 18.01; p = 7.36e-9) groups, there is only weak difference between patient groups (p = 0.04). We added the full IQ as one of the covariates in the statistical analysis and re-performed the group comparisons for the static and dynamic features captured in the main text. The results are displayed in Fig. S2 and Fig. S3. The majority of findings can still hold especially for the dFNC features, indicating that our results are not due to the differences in the overall functioning.


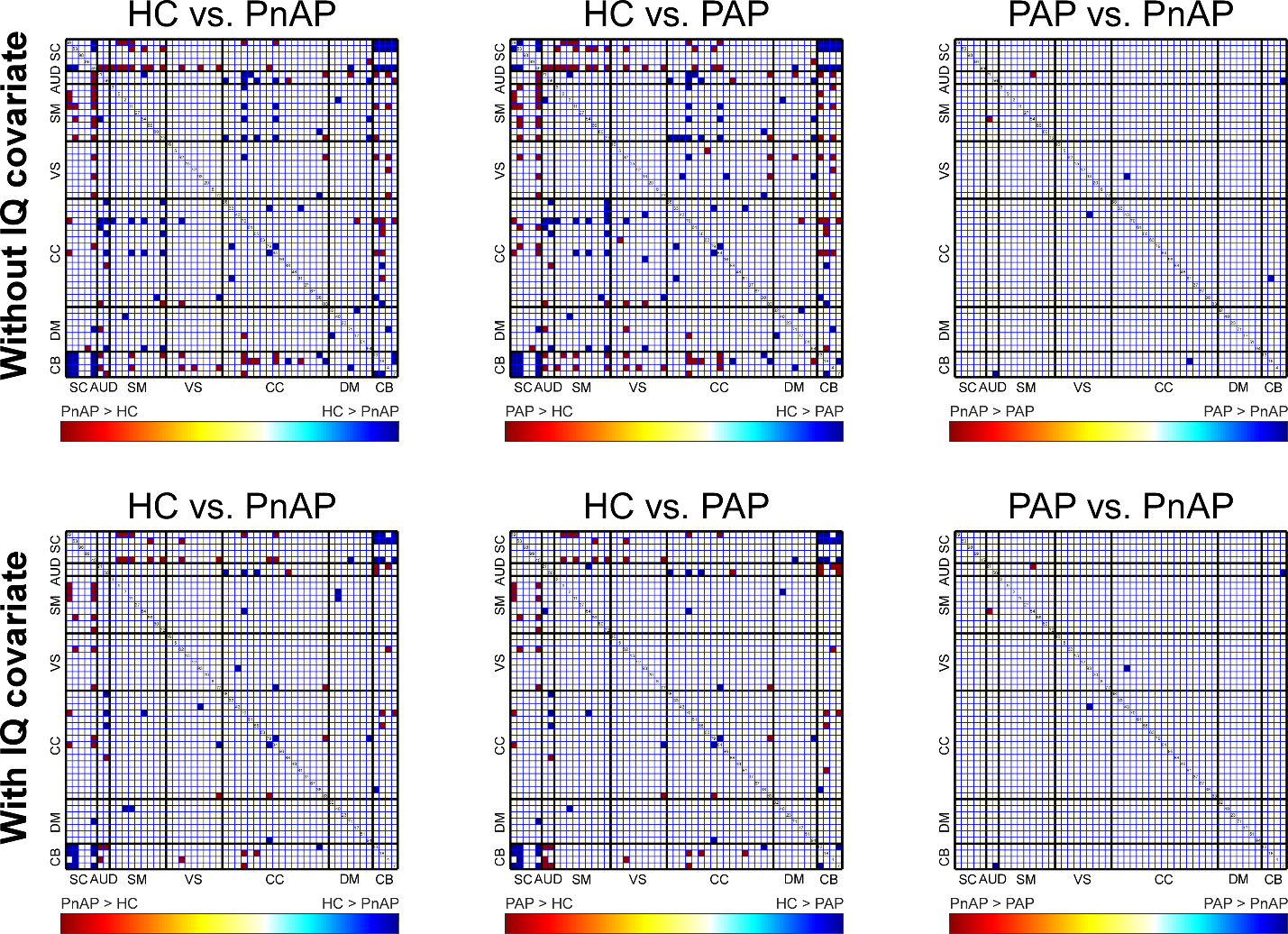


Findings of sFNC analysis with and without full IQ as the covariate.


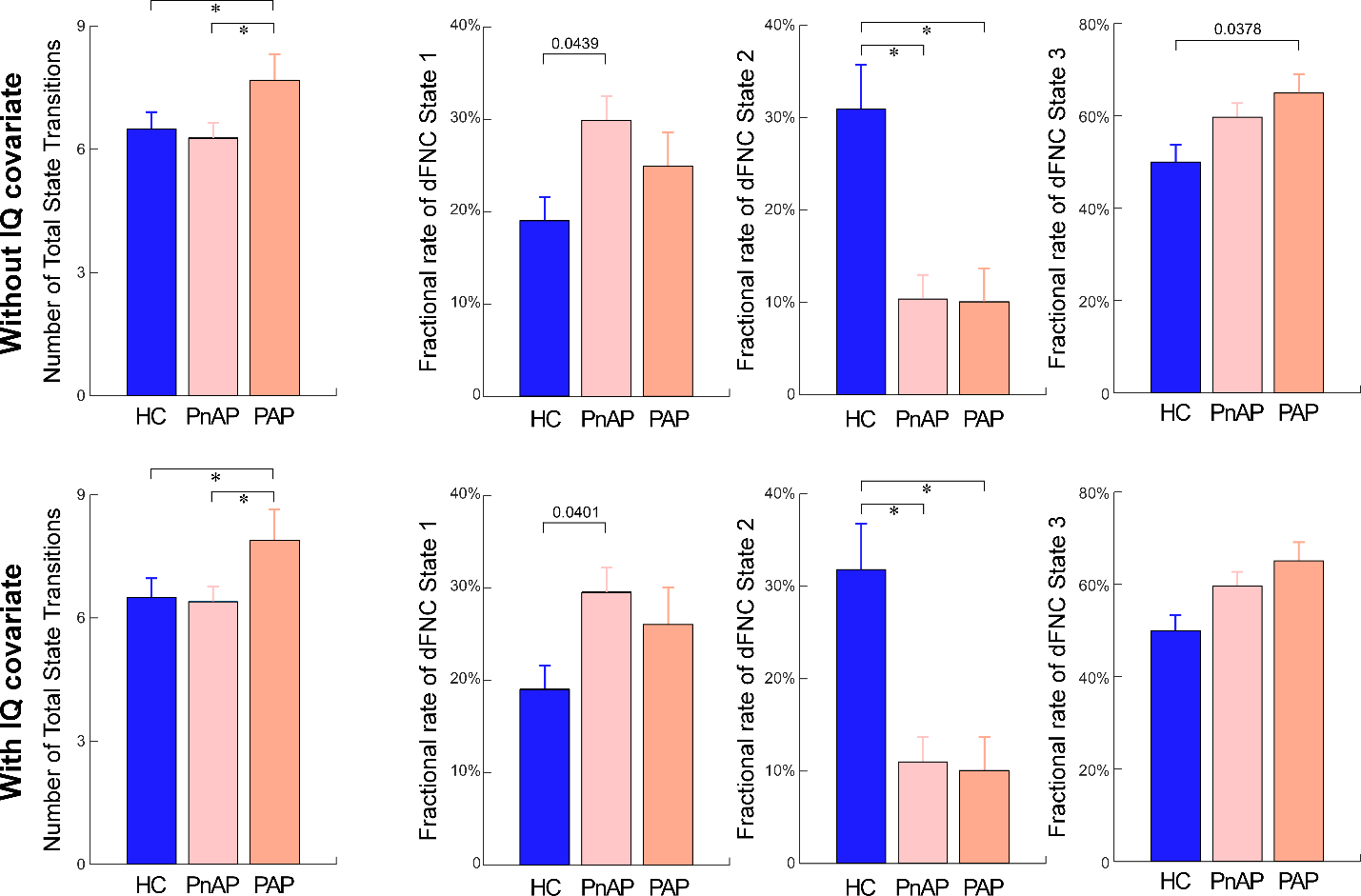


Group differences in dynamic characteristics of dFNC states with and without full IQ as the covariate.

# Associations with Young Mania Rating Scale (YMRS) and Montgomery-Asberg Depression Rating Scale (MADRS)

We performed the correlation analysis between FNC features and YMRS/MADRS to investigate whether FNC features are associated with these clinical profiles. We controlled for age, gender, and site effects and correlated YMRS and MADRS total scores with sFNC features showing significant differences (we also performed the correlation analysis for all dFNC features) for each patient group respectively. It should be noted that only 16 PAPs and 56 PnAPs have YMRS recorded, and 19 PAPs and 50 PnAPs have MADRS recorded. We observed two correlations between sFNC and YMRS total scores. The sFNC between thalamus and CB is negatively correlated with YMRS within PnAP group and the sFNC between MTG and MiFG is negatively correlated with YMRS within PAP group (Fig. S4). There is no significant correlation observed between sFNC and MADRS (p > 0.05) for both groups. Also, dFNC features do not show any significant associations with both YMRS and MADRS.


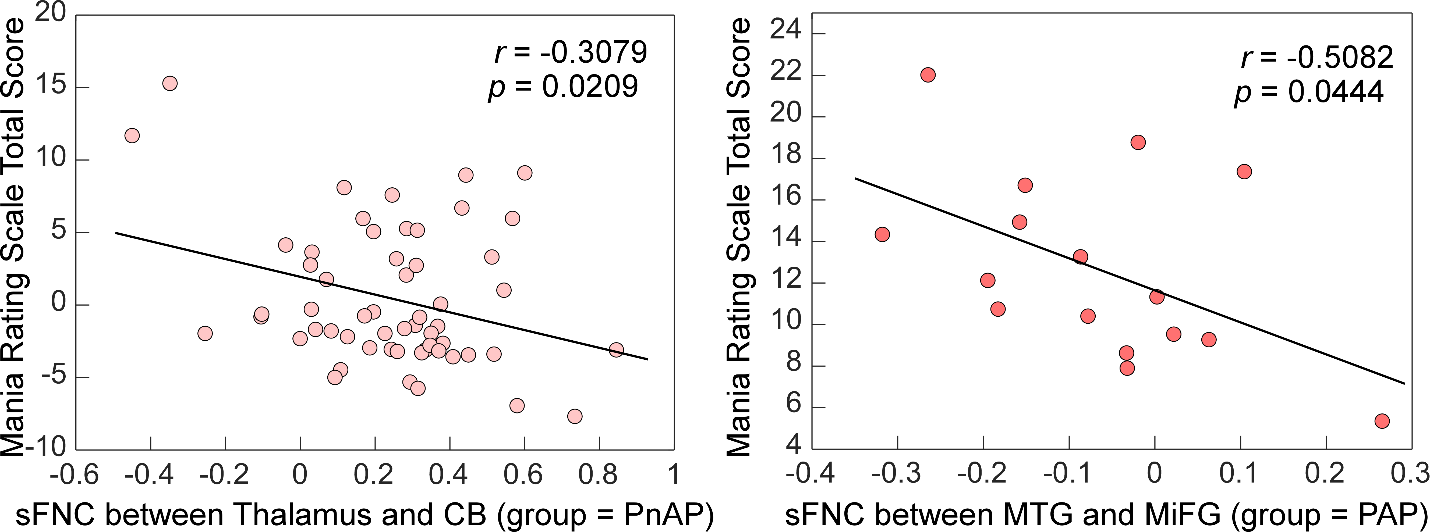


Correlations between sFNC and Young Mania Rating Scale (YMRS) total score.


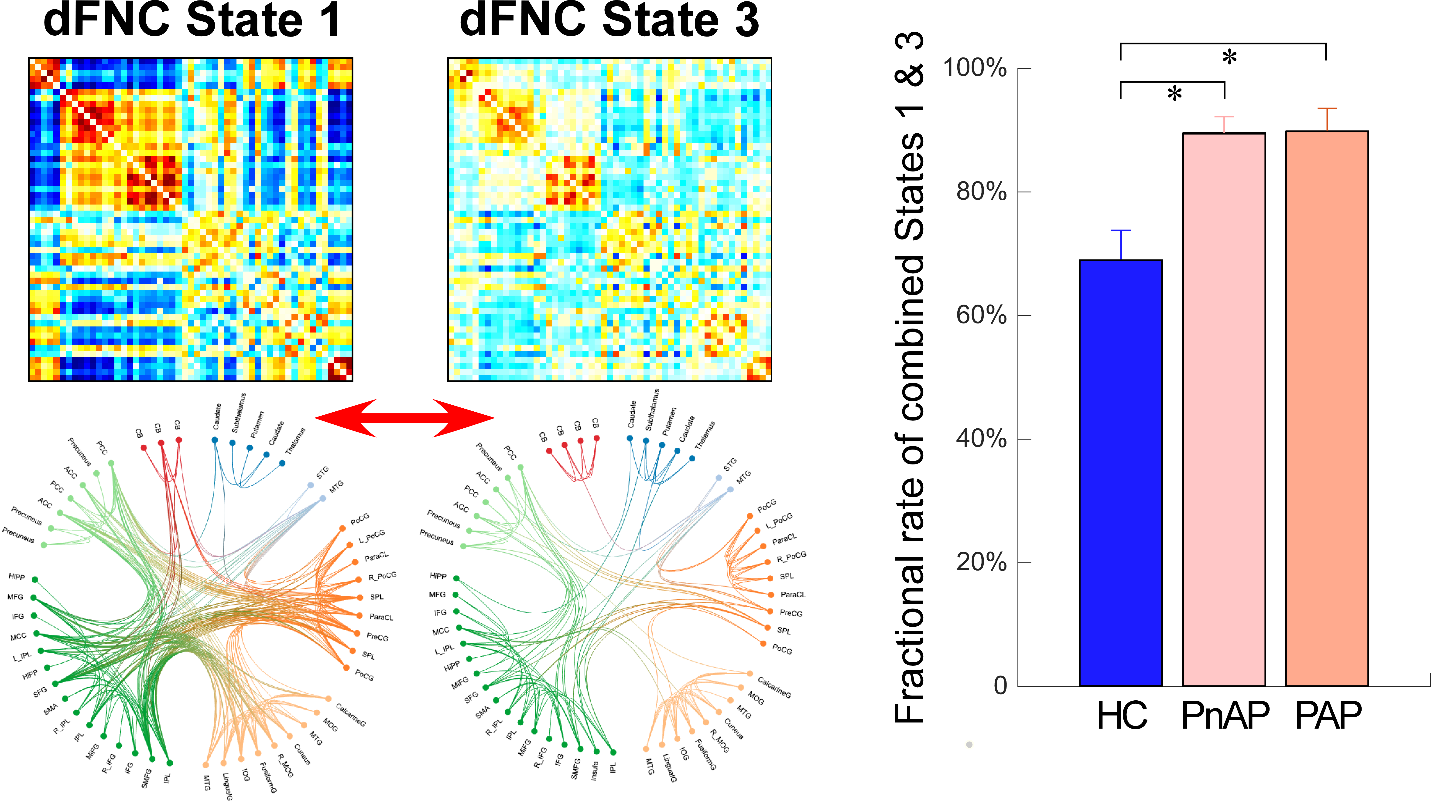


Group difference in the frational rate of combined dFNC states 1 and 3.
